# Parallel Analysis of 124 Universal SNPs for Human Identification by Targeted Semiconductor Sequencing

**DOI:** 10.1038/srep18683

**Published:** 2015-12-22

**Authors:** Suhua Zhang, Yingnan Bian, Zheren Zhang, Hancheng Zheng, Zheng Wang, Lagabaiyila Zha, Jifeng Cai, Yuzhen Gao, Chaoneng Ji, Yiping Hou, Chengtao Li

**Affiliations:** 1 Shanghai Key Laboratory of Forensic Medicine, Institute of Forensic Sciences, Ministry of Justice, P.R. China, Shanghai 200063, P.R. China; 2 State Key Laboratory of Genetic Engineering, Institute of Genetics, School of Life Sciences, Fudan University, Shanghai 200433, P.R. China; 3 Invitrogen Trading (Shanghai) Co., LTD, Shanghai 200050, P.R.China; 4 Department of Forensic Science, School of Basic Medical Sciences, Central South University, Changsha 410013, P.R. China; 5 Department of Forensic Medicine, Medical College of Soochow University, Suzhou 215123, P.R. China; 6 Department of Forensic Genetics, West China School of Preclinical and Forensic Medicine, Sichuan University, Chengdu 610041, P.R.China

## Abstract

SNPs, abundant in human genome with lower mutation rate, are attractive to genetic application like forensic, anthropological and evolutionary studies. Universal SNPs showing little allelic frequency variation among populations while remaining highly informative for human identification were obtained from previous studies. However, genotyping tools target only dozens of markers simultaneously, limiting their applications. Here, 124 SNPs were simultaneous tested using Ampliseq technology with Ion Torrent PGM platform. Concordance study was performed with 2 reference samples of 9947A and 9948 between NGS and Sanger sequencing. Full concordance were obtained except genotype of rs576261 with 9947A. Parameter of F_MAR_ (%) was introduced for NGS data analysis for the first time, evaluating allelic performance, sensitivity testing and mixture testing. F_MAR_ values for accurate heterozygotes should be range from 50% to 60%, for homozygotes or Y-SNP should be above 90%. SNPs of rs7520386, rs4530059, rs214955, rs1523537, rs2342747, rs576261 and rs12997453 were recognized as poorly performing loci, either with allelic imbalance or with lower coverage. Sensitivity testing demonstrated that with DNA range from 10 ng-0.5 ng, all correct genotypes were obtained. For mixture testing, a clear linear correlation (R^2^ = 0.9429) between the excepted F_MAR_ and observed F_MAR_ values of mixtures was observed.

Single nucleotide polymorphisms (SNPs), essentially zero rate of recurrent mutation^1^, are likely in the near future to have a fundamental role in human identification and description. However, currently available dedicated SNP genotyping tools, usually based on the principle of single-base primer extension using commercially available SnapShot chemistry (Life Technologies, San Francisco, CA), only allow the parallel analysis of up to a few dozen SNPs^2^,^3^. Next generation sequencing (NGS), also named Massively parallel sequencing (MPS), and the barcoding system can obtain detailed sequence information of genetic markers and collect massive amounts of data from multiple samples simultaneously^4^,^5^,^6^,^7^,^8^. The libraries of targeted markers in this study were amplified via multi-PCR technology (called Ampliseq) and got sequenced and then aligned to human genome (Hg19). Currently, MiSeq (Illumina) and Ion Torrent Personal Genome Machine (Ion Torrent PGM) (Life Technologies), offer modest set-up and running costs for marker detection, are the most commonly applied for forensic and clinic application^4^,^5^,^8^,^9^. In this study, Ion Torrent PGM which exploits a sensitive semiconductor-based detection of H^+^ ion release during base incorporation onto short template sequences bound to micro-spheres^9^,^10^, was adopted as the studied NGS platform. Ion Torrent PGM is the first commercial sequencing machine that does not require fluorescence and camera scanning, resulting in higher speed, lower cost, and smaller instrument size.

Since universal SNPs showing little allelic frequency variation among populations while remaining highly informative for human identification were obtained from previous studies^11^,^12^,^13^, Life Technologies delivered several beta assays. HID_SNP_v1.0 (containing 103 autosomal SNPs and 33 Y-SNPs), the first beta panel, was tested by Budowle *et al.*^14^. HID_SNP_ v2.2 (containing 136 autosomal SNPs and 33 Y-chromosome markers), the second beta panel for human identification, was first tested by Morling group^15^ and then inter-evaluated by six laboratories^10^. Based on these testing data, 34 upper Y-clade SNPs^13^ and 90 autosomal SNPs^11^,^12^ that have high heterozygosity and a low fixation index (F_ST_), were include in the first commercially available panel named HID-Ion AmpliSeq™ SNP-124. Since no data of this panel has been published yet, we evaluated the panel and explored the application in Chinese HAN population this time.

## Materials and Methods

The main experiments were conducted in Forensic Genetics Laboratory of Institute of Forensic Science, Ministry of Justice, P.R. China, which is an accredited laboratory by ISO 17025, in accordance with quality control measures. All the methods were carried out in accordance with the approved guidelines of Institute of Forensic Sciences, Ministry of Justice, P.R. China.

### Sample preparation

Control DNA of 9947A (Life Technologies) and 9948 (Promega) were adopted as reference samples. Human blood samples involved for study were collected with the approval of Ethics Committee of Institute of Forensic Sciences, Ministry of Justice, P.R. China. Informed consent was obtained for each participant. DNA was extracted using QIAamp® DNA Blood Mini Kit (Qiagen, Hilden, Germany). The quantity of DNA was determined by Quantifiler Human DNA Quantification Kit (Life Technologies) with 7500 Real-time PCR System (Life Technologies).

### Primer Pool for Library Preparation

Primers of 124 universal SNPs (90 autosomal SNPs and 34 upper Y-clade SNPs) were combined in one primer pool. For the 90 auto-SNPs, 43 Ken Kidd SNPs^11^ and 48 SNPforID^12^ (with 1 shared SNP) were included. The other 34 Y-SNPs designated the major haplogroups in the Y-chromosome parsimony tree^13^. The average PCR fragment was 132 bp for the 90 autosomal SNPs and 141 bp for the 34 upper Y-clade SNPs. SNP location information and primer sequences were listed in Supplementary Table S1.

### Library Preparation, Quantification and Emulsion PCR (emPCR)

New technology of Ampliseq and according chemistry of Ion AmpliSeq Library Kit 2.0–96 LV (Life Technologies) were applied for library preparation. Ampliseq technology delivers simple and fast library construction for affordable targeted sequencing of genomic regions. The Ion AmpliSeq™ workflow is based on a transformative technology that simplifies ultrahigh-multiplex PCR amplification and library construction. Utilizing low input DNA, this single-tube workflow is as simple as setting up a PCR reaction and can avoid contamination. The library-PCR system contained 4 μL of 5X Ion AmpliSeq HiFi Master Mix and 10 μL of 2X HID-Ion AmpliSeq™ SNP-124 Panel. Except for the sensitivity and mixture testing of the panel, the initial DNA input was 10 ng for each library construction. The library-PCR parameters were as follows: 2 min at 99°, 18 cycles of 15 s at 99° and 4 min at 60° followed by a 10° hold. For sensitivity testing, 21 cycles were used when DNA amount lower than 1 ng in order to get sufficient amplification. The resulting amplicons were treated with 2 μL FuPa reagent (Life Technologies) to partially digest primers. All libraries were barcoded using Ion Xpress^TM^ Barcode Adapters (Life Technologies). After the ligation with barcodes, libraries were purified with Agencourt AMPure XP Reagents (Beckman Coulter, Brea, CA). Then qPCR methods with Ion Library Quantitation kit (Life Technologies) was adopted for accurate library quantification.

The accurately quantified and diluted pool library was then used to generate template positive Ion Sphere^TM^ Particles (ISP) containing clonally amplified DNA with emPCR technology, which performed on Ion OneTouch2 (OT2) (Life Technologies) by using Ion PGM Template OT2 200 Kit. Quality of emPCR products were evaluated with Ion Sphere^TM^ Quality Control Kit (Life Technologies). The optimal amount of library corresponds to the library dilution point that gives percent of template ISPs between 10–30%. The emPCR products were then enriched on the Ion OneTouch^TM^ ES (Life Technologies).

### Sequencing and Data analysis

NGS was performed on Ion Torrent PGM (Life Technologies) with Ion PGM^TM^ Sequencing 200 Kit v2. Ion Chip types (314, 316 or 318) varied depending on the sample size. Assuming 80% chip loading and 60% usable chip, 8, 38 and 77 samples can been sequenced on 314, 316 and 318 chip. Raw sequencing data were collected as DAT files which were processed on the Ion Torrent Suite Server (v4.0.2). Signal processing, base-calling and barcode de-convolution were performed with Server v4.0.2. HID_SNP_Genotyper.42 (v4.2), Variant Caller (v4.0-r76860) and Coverage Analysis (v4.0-r77897) plug-ins were adopted for data analysis. The reference genome was Hg19.

### Evaluation of HID-Ion AmpliSeq™ SNP-124 Panel

For the concordance and accuracy testing, female sample of 9947A (Life Technology) and male sample of 9948 (Promega) were applied as reference samples. 124 SNPs of the two reference samples were sequenced by NGS and Sanger technologies. Here, Sanger method was adopted for validation of NGS results. For the NGS fragment above 150 bp, same primer pair was used for Sanger sequencing; for the NGS fragment below 150 bp, different primer pair was designed for Sanger sequencing (listed in Supplementary Table S1). For the evaluation of the panel and forensic performance of the 124 SNPs, 45 unrelated healthy Chinese HAN individuals were involved in the study. For the sensitivity testing of the panel, serial dilutions of an In-house male control sample were performed to generate DNA concentrations of 10, 5, 2, 1, 0.5 and 0.2 ng/μL. And 1 μl of each concentration was added in the library PCR-setup system. In other words, the DNA input for sensitivity testing was ranged between 10 ng and 0.2 ng. The libraries of the 6 different concentrations were tested with 314 Ion chip twice. For the mixture study, mixture DNA from control samples of 9947A and 9948 were generated to give ratios of 100:1, 10:1, 5:1, 1:1, 1:5, 1:10 and 1:100. For the 1:1 ratio, 5 ng of each DNA was mixed together. For the ratios of 100:1 and 10:1, 50 pg, 500 pg of 9948 were added to 5 ng of 9947A. For the ratios of 1:10 and 1:100, 500 pg, 50 pg of 9947A were added to 5 ng of 9948. For the 5:1 ratio, 2.5 ng of 9947A was mixed with 500 pg of 9948. Therefore, the DNA input was ranged from 3 ng to 10 ng for library preparation and the 7 libraries of mixtures were tested with 314 Ion chip twice.

## Results and Discussion

### Concordance study

**Figure 1:**
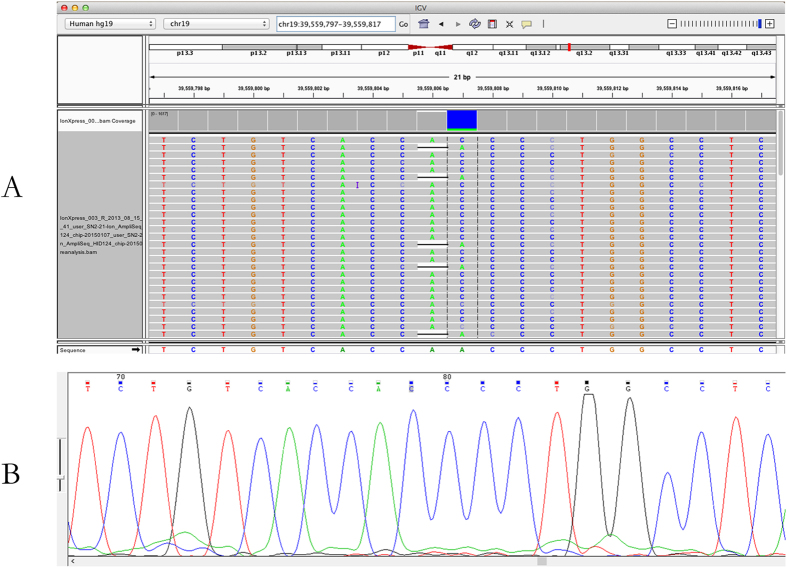
NGS data analyzed with IGV software (A) and Sanger sequencing data analyzed with Chromas (B) at SNP of rs576261 with control sample 9947A.

Control samples of 9947A (Life Technology) and 9948 (Promega) were chosen for concordance study. 124 SNPs (listed in Supplementary Table S1) of these samples were sequenced by NGS and Sanger technologies. HID_SNP_Genotyper.42 (v4.2) plug-in and Chromas were used for the genotyping analysis of NGS data and Sanger sequencing data, respectively. NGS technology has the property of ultra-high throughput but the read length is remarkably short compared to conventional Sanger sequencing. In this study, the shortest PCR length of targeted SNP for NGS is 77 bp and the longest is 244 bp. Thus, for the fragment below 150 bp, different primer pairs were designed for Sanger sequencing (Supplementary Table S1). Sequencing results of 9947A and 9948 by NGS and Sanger sequencing were listed as Supplementary Table S2-1 and Table S2-2, respectively. Except rs576261 (SNP No. 77) of control DNA 9947A (Supplementary Table S2-1), there was complete concordance between the results from NGS and Sanger sequencing of the two reference samples. For SNP rs576261, the NGS results of 9947A was ‘AC’ while the Sanger sequencing result was ‘C’ (Fig. 1). By analyzing BAM file with IGV software, the accurate genotyping at SNP rs576261 of sample 9947A should be ‘C’. The sequence context surrounding SNP rs576261 is TCTGTCACCA[A/C]CCCTGGCCTC. The SNP followed by a homopolymer stretch and a possible allele is identical to the stretch. Misalignment of reads and wrong call of alleles leads to wrong genotyping of NGS (Fig. 1A). And the reads for base A (193) and base C (1422) vary quite significantly.

A parameter of F_MAR_ (Frequency of Major Allele Reads) was adopted here. Analysis with HID_SNP_Genotyper.42 (v4.2) plug-in can provide detail reads at each bases (A, C, G and T) for each SNP. F_MAR_ was calculated as the biggest reads among the four bases dividing the total detected reads. For homozygotes, the optimal F_MAR_ (%) should be equal to 100, while for heterozygotes, the optimal F_MAR_ (%) should be 50. In previous study, Intra-locus balance (the lower peak height dividing the higher peak height for each locus) was applied to measure the balance of heterozygous alleles. According to Eduardoff *et al.*, 50% is a ‘perfect balance’ and 40% threshold (60:40 heterozygote ratio) can give better equilibrium between gaining the highest proportion of reliable genotypes and balanced signals of SNPs. That means the F_MAR_ (%) for accurate heterozygotes should be 50%–60%. And Intra-locus balance above 70% is desired to ensure accurate heterozygote genotyping and to facilitate mixture interpretation for STRs^16^. Here, if the same principle adopted for SNP calling, F_MAR_ (%) for accurate heterozygotes should be ranged from 50% to 59% (1/(0.7 + 1)). Therefore, the boundaries of F_MAR_ (%) was set as 50–60% for ideal allelic-balance of heterozygotes in this study. And according to Eduardoff *et al.*, SNPs with major allelic reads frequencies of 90% or greater were deemed to be homozygous for that allele^2^, as the presence of other bases at a low proportion in the Ion Torrent PGM data arise from non-specific incorporation, but the proportion of a second allele must exceed 10% for Genotyper to call the genotype^10^. For this reason, when F_MAR_ (%) reach >90%, samples cannot be mistyped as heterozygotes. In other words, F_MAR_ (%) values above 90% were recognized as ideal for homozygotes. For the 34 Y-SNPs, only one allele can been detected for each locus. So the F_MAR_ (%) value above 90% is also essential for accurate calling of Y-SNPs. Analyzing the F_MAR_ (%) values of Supplementary Table S2-1, the average F_MAR_ (%) for the detected 33 heterozygotes (except wrong genotype at rs576261) was 52.16. No values of F_MAR_ (%) for heterozygotes was above 60%. While for the F_MAR_ (%) data of Supplementary Table S2-2, three SNP loci (rs7520386, rs214955 and rs4530059) were detected with F_MAR_ (%) values above 60%. For all the detected homozygotes and Y-SNPs, F_MAR_ (%) values above 90%.

### Typing performance of 124 SNPs

**Figure 2:**
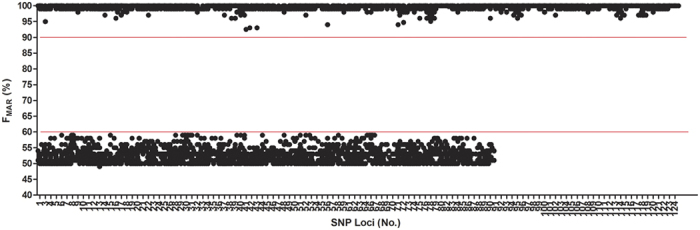
F_MAR_ (%) values of the 124 SNPs among the 45 individuals except rs7520386, rs4530059, rs214955, rs1523537, rs2342747 and rs576261. The SNP loci No. can been found in Supplementary Table S2. The top read line indicated the lowest F_MAR_ (%) values for homozygotes (90%) and the bottom read line indicated the highest F_MAR_ (%) values for heterozygotes (60%).

Above concordance study demonstrated the accuracy rate of SNP typing by PGM sequencing and also suggested several noticeable SNPs. The genotype calling for heterozygote with F_MAR_ above 60% does not equal to wrong calling. To be fully evaluated the Panel, 10 ng initial DNA of 45 unrelated individuals with barcode ‘1–45′ were sequenced on 318 Ion chip twice for intensive study. Identical genotyping results were obtained from the two times of NGS sequencing except at rs7520386 and rs214955 (Supplementary Table S3). Sanger method was adopted for validation of NGS results also. For the wrong genotyping of rs7520386 of sample ‘78#’, abnormal value of F_MAR_ (79.05%) was detected; and for the wrong genotyping of rs214955 of sample ‘A12_045′, lower coverage (<100) was observed. These suggested that attention should be paid to data with lower coverage or abnormal values of F_MAR._ By analyze all the NGS data of the 45 individuals, imbalance of heterozygotes were found at SNPs of rs7520386, rs4530059, rs214955, rs1523537, rs2342747 and rs576261 (Supplementary Table S4). Among the 6 SNPs, SNPs of rs7520386 and rs4530059 showed higher imbalance with mean F_MAR_ (%) values above 60%. Except the 6 SNPs, all the detected F_MAR_ (%) values of the 45 individuals were plotted in Fig. 2. The range of F_MAR_ (%) values were 50%–60% for heterozygotes and 90%–100% for homozygotes and Y-SNPs. And a minimum threshold of 100× coverage was recommended in this study.

There is consistently high coverage with little variation between the samples. However, variation in coverage was observed among the SNPs and each SNP generally showed similarly high or low coverage across the samples. The lower coverage of auto-SNPs were always happened at SNP rs2342747 and rs12997453, which is also observed when genotyping of 9947A and 9948 (Supplementary Table S2-1 and Supplementary Table S2-2). The differences in coverage may primarily be related to differences in PCR amplification efficiency. Modifications of primer concentrations of SNP rs12997453 (lower coverage but ideal performance of heterozygotes) in the pool may provide more library yield. For the SNPs mentioned in Supplementary Table S4, modifications of the primers and/or primer concentrations may provide more balance and higher yield across the SNPs of the panel. Therefore, NGS results of heterozygote SNP with F_MAR_ (%) values above 60% or total coverage below 100x analyzed with HID_SNP_Genotyper.42 (v4.2) plug-in should be checked or discarded. In this study, the 6 SNPs (rs7520386, rs4530059, rs214955, rs1523537, rs2342747 and rs576261) detected with abnormal values of F_MAR_ and SNP of rs2342747, rs12997453 with lower coverage were recognized as poorly performing SNPs. SNP rs2342747 always detected with abnormal values of F_MAR_ and lower coverage maybe should be deleted from the panel.

In the previous Inter-laboratory evaluation of the HID_SNP_ v2.2 with 169-markers for ancestry inference, discordant genotypes detected in 5 SNPs (rs1979255, rs1004357, rs938283, rs2032597 and rs2399332) indicate these loci should be excluded from the panel^10^. Two SNPs of them (rs1979255 and rs938283) were also included in this panel. For the 2 SNPs, the genotyping results of tested samples were correct and the F_MAR_ (%) values were in the range for heterozygotes and homozygotes. Therefore, modification of the primers of ‘problematic SNPs’ may effectively improve the performance of new panel.

### Sensitivity study

Serial dilutions of 10 ng-0.2 ng of a control male DNA sample were made and dilutions were sequenced on Ion 314 chip. Concordant genotyping results were obtained from all these samples except DNA of 0.2 ng at rs2342747 with ‘N/N’. The total reads at this locus was only 20.

**Figure 3:**
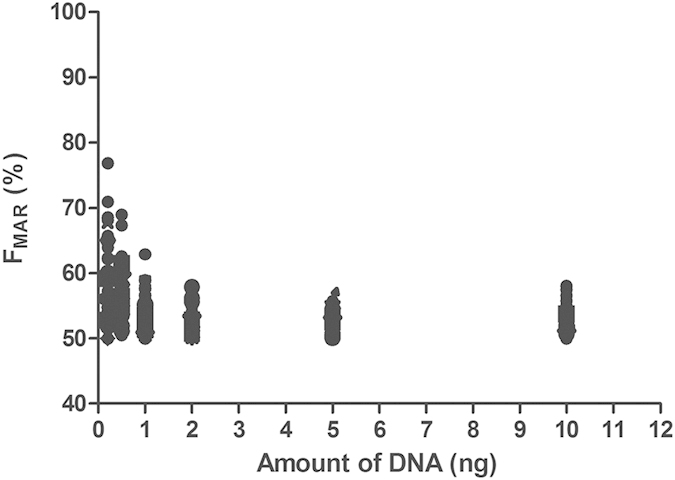
F_MAR_ (%) values of observed heterozygotes with different amount of DNA at the 90 auto-SNPs. 7 poorly performing SNPs (rs7520386, rs4530059, rs214955, rs1523537, rs2342747, rs576261 and rs12997453) were excluded for analysis.

As expected, the allelic balance of heterozygotes varied more in experiments with lower amounts of DNA. The F_MAR_ (%) values of heterozygotes of the 90 auto-SNPs for each dilution were shown in Fig. 3 (except the 7 poorly performing SNPs: rs7520386, rs4530059, rs214955, rs1523537, rs2342747, rs576261 and rs12997453). DNA ranged from 2–10 ng are with optimal F_MAR_ (%) values for heterozygotes. Some terrible F_MAR_ data of heterozygotes were observed when DNA ranged from 0.2–1 ng, especially when DNA below 0.5 ng (Fig. 3). Although NGS data analyzed with correct genotypes by default setting of HID_SNP_Genotyper.42 plug-in for all the called samples, further analysis is essential when data with low coverage or abnormal F_MAR_ values. Above results demonstrated that the optimal amount of DNA in the PCR seemed to be above 0.5 ng, comparable to current STR analysis requirements^16^,^17^. It seems likely that the sensitivity can be improved by further optimization of the primer pool or the PCR or by removing some poor performing SNPs from the panel.

### Mixture study

**Figure 4:**
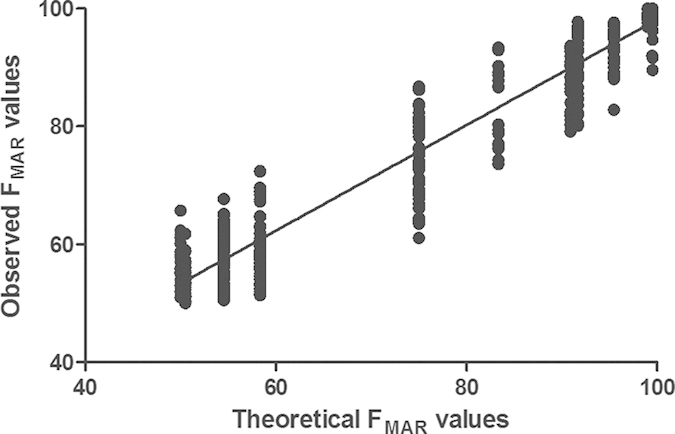
Plotting profiles of values of theoretical F_MAR_ and observed F_MAR_ with different ratios of mixtures. 7 poorly performing SNPs (rs7520386, rs4530059, rs214955, rs1523537, rs2342747, rs576261 and rs12997453) were excluded from the analysis. The correction coefficient of R^2^ is 0.9429.

In this study, mixtures of two reference samples (9947A and 9948) with ratios of 100:1, 10:1, 5:1, 1:1, 1:5, 1:10 and 1:100 were studied. Table 1 listed the theoretical F_MAR_ values of mixtures with all possible genotypes except the two control samples with same genotypes. Figure 4 shows the theoretical F_MAR_ and observed F_MAR_ values of mixtures with genotypes mentioned in Table 1. 7 poorly performing auto-SNPs (rs7520386, rs4530059, rs214955, rs1523537, rs2342747, rs576261 and rs12997453) were excluded from this analysis. There was a clear linear correlation between the excepted and observed F_MA_ values of all the mixtures (R^2^ = 0.9429), which indicated that the assay generated a loyal representation of DNA samples. Detection of mixtures with auto-SNPs is possible by analyzing the F_MAR_ values with NGS data. NGS data can give balanced heterozygous genotypes, providing a more secure basis for analyzing mixtures. It is vital to reliably recognize SNP data as originating from a mixture and not a single profile with the commonly used SnapShot system.

The genotyping results of 34 Y-SNPs of the mixtures were listed in Table S5. 14.71%, 97.06% and 100% of the Y-SNPs were detected in the 100:1, 10:1 and 5:1 mixture of 9947A/9948. For other mixture ratios, 100% of the Y-SNPs were detected.

### Genetic analysis of 124 SNPs in Chinese HAN population

A total of 45 unrelated individuals (17 females and 28 males) of Chinese HAN population were sequenced with the HID-Ion SNP124 panel on Ion 318 chip twice. All the genotypes obtained at the 7 poorly performing SNPs were checked by Sanger sequencing.

Genetic analysis of these 124 SNPs was performed with SNP Analyzer Software^18^. No significant deviation from HWE expectations was detected in the distribution after Bonferroni correction among HAN population (N = 45) of the 90 auto-SNPs. The allelic frequencies and forensic parameters of the 90 auto-SNPs were listed in Table 2. Based on the data of auto-SNPs investigated among HAN individuals, LD analysis was explored. By pairwise LD calculation and Gabriel’s method^18^, the results (Supplementary Fig. S1) shows that no LD was existed among the 90 auto-SNPs. Therefore, the CDP (Cumulative Discrimination Power) was 1–5.2192^−23^ for the 90 auto-SNPs in Chinese HAN. For the Y-SNP analysis, 6 haplotypes were found in the 28 unrelated male individuals. These suggested that the HID-Ion SNP124 panel is suitable for personal identification of HAN population from China.

## Conclusion

NGS plus Ampliseq technology have the capacity to sequence targeted regions of multiple DNA samples with high coverage simultaneously. Compared with Sanger sequencing, this technology also can reduce labor and cost on a per nucleotide bases and indeed on a per sample basis. In this study, with the commercially available SNP panel, high coverage and high throughput of 124 specified targets were detected. The parameter of F_MAR_ (%) was applied for evaluating allelic performance, sensitivity testing and mixture testing, making the NGS data easy to interpret. Further modification of the panel can been explored based on the obtained data. This pilot study of the Ion Torrent PGM Sequencer has demonstrated considerable potential for SNP detection as a low to medium throughput NGS platform. And although capillary electrophoresis remains the gold standard and most cost-effective option for human identification with short tandem repeats (STRs)^16^,^17^, the PGM Sequencer System extends forensic analysis capabilities. SNPs regarding the bio-geographical ancestry (BGA) or externally visible characteristics (EVC) or STRs were explored with PGM also^10^,^15^. These features make markers typing on a NGS platform particularly appealing.

## Additional Information

**How to cite this article**: Zhang, S. *et al.* Parallel Analysis of 124 Universal SNPs for Human Identification by Targeted Semiconductor Sequencing. *Sci. Rep.* **5**, 18683; doi: 10.1038/srep18683 (2015).

## Supplementary Material

Supplementary Information

## Figures and Tables

**Table 1:** Theoretical values of F_MAR_ for different ratios of mixtures (except the two contributors with same genotypes).

Genotype 1	Genotype 2	Mixture	Formular for the calculation of theoretical F_MAR_	Theoretical value of F_MAR_
aa	bb	1:1	2a/(2a + 2b)	0.5000
aa	ab	1:1	3a/(1b + 3a)	0.7500
ab	bb	1:1	3b/(1a + 3b)	0.7500
aa	bb	1:5	10b/(2a + 10b)	0.8333
aa	ab	1:5	7a/(7a + 5b)	0.5833
ab	bb	1:5	11b/(a + 11b)	0.9167
aa	bb	5:1	10a/(10a + 2b)	0.8333
aa	ab	5:1	11a/(11a + b)	0.9167
ab	bb	5:1	7b/(5a + 7b)	0.5833
aa	bb	1:10	20b/(2a + 20b)	0.9091
aa	ab	1:10	12a/(12a + 10b)	0.5455
ab	bb	1:10	21b/(a + 21b)	0.9545
aa	bb	10:1	20a/(20a + 2b)	0.9091
aa	ab	10:1	21a/(21a + b)	0.9545
ab	bb	10:1	12b/(10a + 12b)	0.5455
aa	bb	1:100	200b/(2a + 200b)	0.9901
aa	ab	1:100	102a/(102a + 100b)	0.5050
ab	bb	1:100	201b/(201b + a)	0.9950
aa	bb	100:1	200a/(200a + 2b)	0.9901
aa	ab	100:1	201a/(201a + b)	0.9950
ab	bb	100:1	102b/(102b + 100a)	0.5050

**Table 2:** Allelic frequencies and forensic parameters of 90 auto-SNPs of Chinese HAN population (N = 45).

SNP	Allele	Allele 1	Allele 2	HWE p value	PIC	DP
rs1490413	A/G	0.7220	0.2780	0.3991	0.3209	0.4014
rs7520386	G/A	0.5330	0.4670	0.4723	0.3739	0.4978
rs4847034	G/A	0.5110	0.4890	0.4578	0.3749	0.4998
rs560681	A/G	0.5670	0.4330	0.0362	0.3705	0.4910
rs10495407	G/A	0.7330	0.2670	0.5384	0.3148	0.3914
rs891700	A/G	0.5440	0.4560	0.4210	0.3731	0.4961
rs1413212	C/T	0.5220	0.4780	0.3019	0.3745	0.4990
rs876724	T/C	0.5220	0.4780	0.0506	0.3745	0.4990
rs1109037	G/A	0.6220	0.3780	0.3170	0.3597	0.4702
rs993934	A/G	0.6220	0.3780	0.7889	0.3597	0.4702
rs12997453	G/A	0.6110	0.3890	0.2574	0.3624	0.4754
rs907100	C/G	0.5560	0.4440	0.5023	0.3718	0.4937
rs1357617	T/A	0.7440	0.2560	0.7751	0.3084	0.3809
rs4364205	G/T	0.5780	0.4220	0.5502	0.3688	0.4878
rs1872575	A/G	0.5670	0.4330	0.3467	0.3705	0.4910
rs1355366	T/C	0.8440	0.1560	0.5787	0.2287	0.2633
rs6444724	T/C	0.6440	0.3560	0.8396	0.3534	0.4585
rs2046361	T/A	0.5220	0.4780	0.8708	0.3745	0.4990
rs6811238	G/T	0.7000	0.3000	0.6361	0.3318	0.4200
rs1979255	G/C	0.5000	0.5000	0.4561	0.3750	0.5000
rs717302	A/G	0.9110	0.0890	0.7982	0.1490	0.1622
rs159606	G/A	0.5780	0.4220	0.2166	0.3688	0.4878
rs7704770	A/G	0.6560	0.3440	0.8230	0.3495	0.4513
rs251934	A/G	0.9110	0.0890	0.7037	0.1490	0.1622
rs338882	A/G	0.5780	0.4220	0.9892	0.3688	0.4878
rs13218440	G/A	0.5670	0.4330	0.1369	0.3705	0.4910
rs214955	T/C	0.6000	0.4000	0.9011	0.3648	0.4800
rs727811	T/G	0.6220	0.3780	0.3170	0.3597	0.4702
rs6955448	C/T	0.7670	0.2330	0.9163	0.2935	0.3574
rs917118	C/T	0.7220	0.2780	0.4225	0.3209	0.4014
rs321198	C/T	0.5780	0.4220	0.2166	0.3688	0.4878
rs737681	C/T	0.8440	0.1560	0.6808	0.2287	0.2633
rs10092491	C/T	0.6780	0.3220	0.9244	0.3413	0.4366
rs4288409	C/A	0.6330	0.3670	0.9744	0.3567	0.4646
rs2056277	C/T	0.9330	0.0670	0.4136	0.1172	0.1250
rs1015250	G/C	0.5890	0.4110	0.7092	0.3670	0.4842
rs7041158	C/T	0.7000	0.3000	0.6361	0.3318	0.4200
rs1463729	C/T	0.5670	0.4330	0.7847	0.3705	0.4910
rs1360288	C/T	0.7220	0.2780	0.4225	0.3209	0.4014
rs10776839	T/G	0.5000	0.5000	0.0526	0.3750	0.5000
rs826472	C/T	0.8330	0.1670	0.8196	0.2395	0.2782
rs735155	T/C	0.7890	0.2110	0.5909	0.2775	0.3330
rs3780962	A/G	0.5000	0.5000	0.8815	0.3750	0.5000
rs740598	A/G	0.6000	0.4000	0.9011	0.3648	0.4800
rs964681	T/C	0.7000	0.3000	0.2682	0.3318	0.4200
rs1498553	T/C	0.5110	0.4890	0.6522	0.3749	0.4998
rs901398	T/C	0.7220	0.2780	0.1229	0.3209	0.4014
rs10488710	G/C	0.6220	0.3780	0.7141	0.3597	0.4702
rs2076848	A/T	0.6110	0.3890	0.0451	0.3624	0.4754
rs2269355	G/C	0.5000	0.5000	0.4561	0.3750	0.5000
rs2111980	T/C	0.6000	0.4000	0.4561	0.3648	0.4800
rs10773760	A/G	0.6000	0.4000	0.2636	0.3648	0.4800
rs1335873	A/T	0.7670	0.2330	0.0887	0.2935	0.3574
rs1886510	G/A	0.9220	0.0780	0.6611	0.1335	0.1438
rs1058083	G/A	0.5780	0.4220	0.2166	0.3688	0.4878
rs354439	A/T	0.5440	0.4560	0.6911	0.3731	0.4961
rs1454361	A/T	0.5440	0.4560	0.4210	0.3731	0.4961
rs722290	C/G	0.5560	0.4440	0.9465	0.3718	0.4937
rs873196	T/C	0.8560	0.1440	0.6361	0.2161	0.2465
rs4530059	G/A	0.6890	0.3110	0.8454	0.3367	0.4286
rs2016276	T/C	0.6220	0.3780	0.3671	0.3597	0.4702
rs1821380	C/G	0.5670	0.4330	0.7385	0.3705	0.4910
rs1528460	T/C	0.5890	0.4110	0.1086	0.3670	0.4842
rs729172	G/T	0.8780	0.1220	0.7303	0.1913	0.2142
rs2342747	G/A	0.7110	0.2890	0.7834	0.3265	0.4110
rs430046	C/T	0.6780	0.3220	0.1189	0.3413	0.4366
rs1382387	A/C	0.6330	0.3670	0.1882	0.3567	0.4646
rs9905977	G/A	0.6000	0.4000	0.0183	0.3648	0.4800
rs740910	A/G	0.9220	0.0780	0.5637	0.1335	0.1438
rs938283	T/C	0.8890	0.1110	0.8272	0.1779	0.1974
rs2292972	T/C	0.5560	0.4440	0.5915	0.3718	0.4937
rs1493232	C/A	0.6110	0.3890	0.9029	0.3624	0.4754
rs9951171	G/A	0.5330	0.4670	0.9047	0.3739	0.4978
rs1736442	C/T	0.5780	0.4220	0.5322	0.3688	0.4878
rs1024116	C/T	0.8440	0.1560	0.6808	0.2287	0.2633
rs719366	A/G	0.7670	0.2330	0.3397	0.2935	0.3574
rs576261	A/C	0.5330	0.4670	0.6318	0.3739	0.4978
rs1031825	C/A	0.5220	0.4780	0.4472	0.3745	0.4990
rs445251	C/G	0.6330	0.3670	0.0502	0.3567	0.4646
rs1005533	G/A	0.6000	0.4000	0.9011	0.3648	0.4800
rs1523537	T/C	0.5780	0.4220	0.2268	0.3688	0.4878
rs722098	G/A	0.5330	0.4670	0.2810	0.3739	0.4978
rs2830795	A/G	0.5220	0.4780	0.6637	0.3745	0.4990
rs2831700	A/G	0.5220	0.4780	0.3019	0.3745	0.4990
rs914165	G/A	0.6560	0.3440	0.8230	0.3495	0.4513
rs221956	C/T	0.5560	0.4440	0.5915	0.3718	0.4937
rs733164	G/A	0.8780	0.1220	0.0088	0.1913	0.2142
rs987640	A/T	0.5220	0.4780	0.6637	0.3745	0.4990
rs2040411	G/A	0.7780	0.2220	0.4808	0.2858	0.3454
rs1028528	A/G	0.6670	0.3330	0.5023	0.3456	0.4442

## References

[b1] Reich D. E. *et al.* Human genome sequence variation and the influence of gene history, mutation and recombination. Nat Genet. 32, 135–42 (2002). 12161752 10.1038/ng947

[b2] Daniel R. *et al.* A SNaPshot of next generation sequencing for forensic SNP analysis. Forensic Sci Int Genet. 14, 50–60 (2015). 25282603 10.1016/j.fsigen.2014.08.013

[b3] Ralf A., van O. M., Zhong K. & Kayser M. Simultaneous analysis of hundreds of Y-chromosomal SNPs for high-resolution paternal lineage classification using targeted semiconductor sequencing. Hum Mutat. 36, 151–9 (2015). 25338970 10.1002/humu.22713

[b4] Flicek P. & Birney E. Sense from sequence reads: methods for alignment and assembly. Nature methods. 6, S6–S12 (2009). 19844229 10.1038/nmeth.1376

[b5] Andersen J. D. *et al.* Next-generation sequencing of multiple individuals per barcoded library by deconvolution of sequenced amplicons using endonuclease fragment analysis. Biotechniques. 57, 91–94 (2014). 25109295 10.2144/000114200

[b6] Allegue C. *et al.* Genetic Analysis of Arrhythmogenic Diseases in the Era of NGS: The Complexity of Clinical Decision-Making in Brugada Syndrome. PLoS One. 10, e0133037 (2015). 26230511 10.1371/journal.pone.0133037 PMC4521779

[b7] Børsting C. & Morling N. Next generation sequencing and its applications in forensic genetics. Forensic Sci Int Genet. 15, S1872–4973 (2015). 10.1016/j.fsigen.2015.02.002 25704953

[b8] Geppert M. *et al.* Identification of new SNPs in native South American populations by resequencing the Y chromosome. Forensic Sci Int Genet. 15, 111–114 (2015). 25303787 10.1016/j.fsigen.2014.09.014

[b9] Merriman B., Ion Torrent R&Team D & Rothberg J. M. Progress in Ion Torrent semiconductor chip based sequencing. Electrophoresis. 33, 3397–3417 (2012). 23208921 10.1002/elps.201200424

[b10] Eduardoff M. *et al.* Inter-laboratory evaluation of SNP-based forensic identification by massively parallel sequencing using the Ion PGM^TM^. Forensic Sci Int Genet. 17, 110–121 (2015). 25955683 10.1016/j.fsigen.2015.04.007

[b11] Kidd K. K. *et al.* Developing a SNP panel for forensic identification of individuals. Forensic Sci Int. 164, 20–32 (2006). 16360294 10.1016/j.forsciint.2005.11.017

[b12] Musgrave-Brown E. *et al.* Forensic validation of the SNPforID 52-plex assay. Forensic Sci Int Genet. 1, 186–90 (2007). 19083753 10.1016/j.fsigen.2007.01.004

[b13] Karafet T. M. *et al.* New binary polymorphisms reshape and increase resolution of the human Y chromosomal haplogroup tree. Genome Res. 18, 830–838 (2008). 18385274 10.1101/gr.7172008 PMC2336805

[b14] Seo S. B. *et al.* Single nucleotide polymorphism typing with massively parallel sequencing for human identification. Int J Legal Med. 127, 1079–1086 (2013). 23736940 10.1007/s00414-013-0879-7

[b15] Børsting C., Fordyce S. L., Olofsson J., Mogensen H. S. & Morling N. Evaluation of the Ion Torrent™ HID SNP 169-plex: A SNP typing assay developed for human identification by second generation sequencing. Forensic Sci Int Genet. 12, 144–154 (2014). 24997319 10.1016/j.fsigen.2014.06.004

[b16] Collins P. J. *et al.* Developmental Validation of a Single-Tube Amplification of the 13 CODIS STR Loci, D2S1338, D19S433, and Amelogenin: The AmpFl STR Identifiler PCR Amplification Kit. J Forensic Sci. 49, 1265–77 (2004). 15568700

[b17] Mönich U. J. *et al.* Probabilistic characterisation of baseline noise in STR profiles. Forensic Sci Int Genet. 19, 107–122 (2015). 26218981 10.1016/j.fsigen.2015.07.001

[b18] Yoo J., Lee Y., Kim Y., Rha S. Y. & Kim Y. SNPAnalyzer 2.0: a web-based integrated workbench for linkage disequilibrium analysis and association analysis. BMC Bioinformatics. 9, 290 (2008). 18570686 10.1186/1471-2105-9-290 PMC2453143

