# Supplementary material for: Parallel Analysis of 124 Universal SNPs for Human Identification by Targeted Semiconductor Sequencing
Source: Sci Rep. 2015 Dec 22;5:18683. doi: 10.1038/srep18683 (PMC4687036; doi:10.1038/srep18683)
Supplement: Supplementary Information [file srep18683-s1.pdf]

Supplementary Figures S1 and Supplementary Tables S1-S5

Parallel Analysis of 124 universal SNPs for human identification by Targeted Semiconductor Sequencing

Suhua Zhang[1,2], Yingnan Bian[1], Zheren Zhang[3], Hancheng Zheng[3], Zheng Wang[1], Lagabaiyila Zha[4], Jifeng Cai[4], Yuzhen Gao[5], Chaoneng Ji[2], Yiping Hou[6], Chengtao Li[1]*

[1] Shanghai Key Laboratory of Forensic Medicine, Institute of Forensic Sciences, Ministry of Justice, P.R. China, Shanghai 200063, P.R. China

[2] State Key Laboratory of Genetic Engineering, Institute of Genetics, School of Life Sciences, Fudan University, Shanghai 200433, P.R. China

[3] Invitrogen Trading (Shanghai) Co., LTD

[4] Department of Forensic Science, School of Basic Medical Sciences, Central South University, Changsha 410013, P.R. China

[5] Department of Forensic Medicine, Medical College of Soochow University, Suzhou 215123, P.R. China

[6] Department of Forensic Genetics, West China School of Preclinical and Forensic Medicine, Sichuan University, Chengdu 610041, P.R.China

***** Corresponding author: Chengtao Li.

Shanghai Key Laboratory of Forensic Medicine, Institute of Forensic Sciences, Ministry of Justice, P.R. China, Shanghai 200063, P.R. China

E-mail: lichengtaohla@163.com; Phone: +86-21-52351327; Fax: +86-21-52352959.

Supplementary Table S1 Detail information of 124 SNPs included in HID-Ion AmpliSeq™ SNP-124 Panel

SNP	Chromosome	Position (GRch37.p10)	Ion_AmpliSeq_Fwd_Primer	Ion_AmpliSeq_Rev_Primer	NGS Library length (bp)	Sanger sequencing_Fwd_Primer	Sanger sequencing_Rev_Primer
L298	Y	8467290	GGATACTTGCAGGCAGGCTT	TGCCAGGCTATCAGAGCTGT	97	CCCAATTATTTCACGCAACC	AAAGCGGCCTGATATCTGAA
M479	Y	20834667	CCTTTGCTCATTTTCCCCTTCCA	TCCATCAAGGCCTTGGGTAG	76	CCAGCACACAACACTGGACT	GGTGGTGGAAGATGGAAGTG
P202	Y	14001024	TGGCAAGCCCAGGATAGAGA	TGTCACAAAACCATCTGCTTTGTATT	184	Same with Ion_AmpliSeq_Fwd_Primer	Same with Ion_AmpliSeq_Rev_Primer
P256	Y	8685230	AGTTCTTGGTTTTCCCATTGACC	GCAGAAGTTCTGAGGAAAAGGAAA	119	TTGAACCCATCCTCTGCTTC	TTAGCAGCTTTGGGAAGGAA
rs1005533	20	39487110	TGTGGGTTTGTGTGTGAGTGT	GCTGGGTGAGGAGTGCAG	173	Same with Ion_AmpliSeq_Fwd_Primer	Same with Ion_AmpliSeq_Rev_Primer
rs10092491	8	28411072	CCTGGATACCTCTGGGCCATA	CCCCTGAGAGTTTTAACCAGTTTCA	166	Same with Ion_AmpliSeq_Fwd_Primer	Same with Ion_AmpliSeq_Rev_Primer
rs1015250	9	1823774	AGGGTAAAAGGTTACTAAGTGATGGAGT	CATAAGACATTAGGTGGATTCATAGCTGTTTA	114	TCAATAACAATAGTTCCCTG	TCATAAGACATTAGGTGGA
rs1024116	18	75432386	GGGAAGTGGAACATGTACAGGAAA	GAAGTTCAATAACGTCCAGGGAGT	169	Same with Ion_AmpliSeq_Fwd_Primer	Same with Ion_AmpliSeq_Rev_Primer
rs1028528	22	48362290	CTTGGCAAGCACAATGCAAAAG	GAGGATGAAGGTTAGAGCCAGAC	86	TGCTGTACTTGGGATTGA	AGGGAGGATGAAGGTTAG
rs1031825	20	4447483	GTGAGCCCATGCTGCCTTAT	CTATGCCTTTAAGTGCCAAGCC	158	Same with Ion_AmpliSeq_Fwd_Primer	Same with Ion_AmpliSeq_Rev_Primer
rs10488710	11	115207176	TGCGAAACATGCTTCAGAGGTTATAAAAGAC	AGTGTTTTAGTAATAGAGGTTTTACTGTATTAGG	135	AGGGTAGAAACCAAGAGT	CTGTATGTGGAGGTGAAG
rs10495407	1	238439308	CTTGACAATTTATGCCTGCAGGTTT	GTTCTCCCAAATTTACATTGCCACT	173	Same with Ion_AmpliSeq_Fwd_Primer	Same with Ion_AmpliSeq_Rev_Primer
rs1058083	13	100038233	ACTGGTTCAAGGTTCTGGAGTT	AGGAAGCCTGCTGTTGTTCT	118	CTGGGAGTGAGGGAGAAA	AGGAAGCCTGCTGTTGTT
rs10773760	12	130761696	CGTCGGGACCAGCTTCTG	GACACCGAGTGTCTTGTACATTCC	96	ATTCCTTCTACTACCTCCAA	ACAGCCATCACTGTCCAT
rs10776839	9	137417308	CTGCGAAATCCCAAAATGCCA	CCTGTTGACCCCGTCGTA	162	Same with Ion_AmpliSeq_Fwd_Primer	Same with Ion_AmpliSeq_Rev_Primer
rs1109037	2	10085722	TGTCACAGAGCTGGTGGTGA	GGGTCTCAAGGTCGTGCCA	108	TACACTCCCTGCCTCCCA	CCAATTCCTCCACATCCTTGTTGGT
rs12997453	2	182413259	CTAGGACCTGTAAGAGTCTGTGATTCTA	TTAACAGCTCTGATGATGTGCAAGA	175	Same with Ion_AmpliSeq_Fwd_Primer	Same with Ion_AmpliSeq_Rev_Primer
rs13218440	6	12059954	CTGCTGTGGACTGAAACTTGATC	GTACCTCAAGCAGCCCCAA	99	CTGGAATTACAGGCGTGAG	TCAGAGGCAGTGGGAGCT
rs1335873	13	20901724	ATTCAACAAACGTGTGATGCTCT	GCACGTGGATGATATGGTTTCTCAA	115	CGTGGATGATATGGTTTC	TTCTGTGGGTGCTCTGTC
rs13447443	Y	22739301	GGATTTGTTTAAAGAGGAAGTGTGGATT	CCAAACCCTGATCATTCCCCTTC	173	Same with Ion_AmpliSeq_Fwd_Primer	Same with Ion_AmpliSeq_Rev_Primer
rs1355366	3	190806108	GTATCACCATCCAGCTGGCA	CTAAATGCTCCTGCACCAAAATTACA	167	Same with Ion_AmpliSeq_Fwd_Primer	Same with Ion_AmpliSeq_Rev_Primer
rs1357617	3	961782	GTCTCAAACGCCATCAGTATAGGT	CCATTCCTCATTGGCAGCTGA	175	Same with Ion_AmpliSeq_Fwd_Primer	Same with Ion_AmpliSeq_Rev_Primer

SNP	Chromosome	Position (GRch37.p10)	Ion_AmpliSeq_Fwd_Primer	Ion_AmpliSeq_Rev_Primer	NGS Library length (bp)	Sanger sequencing_Fwd_Primer	Sanger sequencing_Rev_Primer
rs1360288	9	128968063	CTCTCTGTGTGTGGCTTTGG	GCCTCCATCTTTCAGGTCTGG	120	TCTGGGGAGTAGAGGAGAC	AAGCCTCCATCTTTCAGGTC
rs1382387	16	80106361	CTGGAGTACTTAATAAGACGCTGCAT	TCATTCCCATGTTGTGTACACGAAA	99	ACGGCACGAAGGAGAAAC	CCGCTCCCTCCATAAGAG
rs1413212	1	242806797	GCAACATTCCATTATCCAGGAGACA	GGTCAACAACCTCCTTTGGAAACA	92	CACTCTTCTGAATCCTGGTC	AGCCTCATAGGGTTTGCT
rs1454361	14	25850832	TGTCCATCATCAGTAAGACACTTTTCAG	AAAACCACCATCTCCAGCAAGT	126	GGGAGGAGGGAAATACAC	AATGAAGTTGCACGCAGA
rs1463729	9	126881448	GCATTGTCTCGTGTGTTTCACAT	GTCTCTGCCCTTATTCTGGCTT	98	TGTGTGCATTGTCTCGTGTG	TTCTGGCTTTGGCAGCATAC
rs1490413	1	4367323	GCTCAGCACAGAAATAAAAGCTCTT	TGTTGGTGGAAGGGACTGAGA	175	Same with Ion_AmpliSeq_Fwd_Primer	Same with Ion_AmpliSeq_Rev_Primer
rs1493232	18	1127986	TCTCAAGGAATTAATCACCAAAGCTATTCT	TTGGTTTTAGATGTGTCTCAAACTGTTTATT	100	ACGAGCCTATCAAAGAGC	CATGACCAGCCAAGATTA
rs1498553	11	5709028	ACTACTGTTTTCTCTCAGCTGCAATT	AGAGACCTGTTCTCTGTCCATTATTTCT	162	Same with Ion_AmpliSeq_Fwd_Primer	Same with Ion_AmpliSeq_Rev_Primer
rs1523537	20	51296162	GCTTAGGGTCTTAATACATTCATTTCTGC	ACTGGGTGAGACAATGCACA	120	AGTCTCCCTCAGGAACAG	AATCAGCCAATCCATACC
rs1528460	15	55210705	GTGAAACTCAGCAAACTGCATCT	GAGCTAAGAGAGATTGATTATGTTGGGA	171	Same with Ion_AmpliSeq_Fwd_Primer	Same with Ion_AmpliSeq_Rev_Primer
rs159606	5	17374898	TGTGAGCACAAACATCTAATAAGCACTT	TGATCCACATTGTATGGTTTTTAGGCA	160	Same with Ion_AmpliSeq_Fwd_Primer	Same with Ion_AmpliSeq_Rev_Primer
rs16980426	Y	22214221	AGACAAATGCCAGAAGAGCAAATAC	TGCCAGTAGCTTTGTGTTTTCAGTA	123	TGCCAGAAGAGCAAATACGG	GTAGCACCTACACTAAGTTC
rs16981290	Y	7568568	GTCTGAGCTTGGGCAGAATCTA	CTTACCTGGTCTTCATCTGATTCACC	161	Same with Ion_AmpliSeq_Fwd_Primer	Same with Ion_AmpliSeq_Rev_Primer
rs17222573	Y	17891241	TCACAAGCAGCCTCTGAATCC	TGTGCCTTACCCGTGTCTGA	112	TCTATGAATGCCTCACAACC	TTCTGTATTAGCCTCCAGCC
rs17250845	Y	8418927	CTTGCCGTCATCACAGATGG	GCCTTGCAGGGGCTTTCT	94	CCTCAACAGCACCACTGAAA	CAAACCTGAAAACCCAGAGG
rs17269816	Y	17053771	AAGACTACATTTTCTAACATAATACCAGCCAA	ACACCAGTCTACATTTTCCAGGAG	120	AGACTGGGCCAAATCTCC	CCTTGTATTCCACCTCAT
rs17306671	Y	14031334	CTACATGCACACACAAACACATTCT	GTTCATGTCCTATGTGGCATGGT	175	Same with Ion_AmpliSeq_Fwd_Primer	Same with Ion_AmpliSeq_Rev_Primer
rs1736442	18	55225777	TCCTGAGTCTTAGCAAACTGCAA	AGTGGGACAGTTAAGAGAAGGCT	168	Same with Ion_AmpliSeq_Fwd_Primer	Same with Ion_AmpliSeq_Rev_Primer
rs17842518	Y	23443971	TGTGCTCAGATCTGCATTGGA	ATGCCCTTCTCTCCATGTCC	89	AGGGAAAGGAAGAGTAAAG	CACAGTAATGGGAGTGAAG
rs1821380	15	39313402	GAGCACTCTTTCCTTGTCCCTT	CAGTGCAAGACAAGCGATTGA	171	Same with Ion_AmpliSeq_Fwd_Primer	Same with Ion_AmpliSeq_Rev_Primer
rs1872575	3	113804979	CCCACATGAGGGTGAAGAGAT	CATCACTCCCACTTCAAGGCT	174	Same with Ion_AmpliSeq_Fwd_Primer	Same with Ion_AmpliSeq_Rev_Primer
rs1886510	13	22374700	CACAAGGGCGTCCCCATTTA	AGTCTCCAAACCGCTGTAATATTTTTGA	165	Same with Ion_AmpliSeq_Fwd_Primer	Same with Ion_AmpliSeq_Rev_Primer
rs1979255	4	190318080	GAATCATAGCTTGTGTTGGTCAGG	GGATGGTATTTAGGTCAAATGAATTTCCA	100	TTCCACGAAAGTTCTTCTCC	ATAGCTTGTGTTGGTCAGGG
rs2016276	15	24571796	GGAAATTGTACCTTGCCACTTTGT	GTATTTGCATCCCAGCCTCCAC	99	TATGTCATCATCTGGCTCA	AGTAAGTTTGCTGCTGTTA

SNP	Chromosome	Position (GRch37.p10)	Ion_AmpliSeq_Fwd_Primer	Ion_AmpliSeq_Rev_Primer	NGS Library length (bp)	Sanger sequencing_Fwd_Primer	Sanger sequencing_Rev_Primer
rs20320	Y	14898163	AGTCTGTGTCTTTTTCCTTTGCAGA	GGTGGACTAAGTTTGCCTTCTCC	100	TGCAGATAGAACAGCTGTAG	GGTGGACTAAGTTTGCCTTC
rs2032595	Y	14813991	TGCTTTTTGTTTTGCAGAGAGCTT	GGTAATCTCATAGGTCTCTGACTGTTCA	175	Same with Ion_AmpliSeq_Fwd_Primer	Same with Ion_AmpliSeq_Rev_Primer
rs2032599	Y	14851554	GGCAGGAAAACATGAAGCCATTG	TCATCAGAGATTCTTCCATGAAAATGT	242	Same with Ion_AmpliSeq_Fwd_Primer	Same with Ion_AmpliSeq_Rev_Primer
rs2032602	Y	14954280	CGTTCTTCTCCGTCACAGCAAA	ACAAGACCCATCTTGCAAGGAA	175	Same with Ion_AmpliSeq_Fwd_Primer	Same with Ion_AmpliSeq_Rev_Primer
rs2032624	Y	15026424	CACAGTACTCACTTTAGGTTTGCCATA	CTGCAGTTTTCCCAGATCCTGA	173	Same with Ion_AmpliSeq_Fwd_Primer	Same with Ion_AmpliSeq_Rev_Primer
rs2032631	Y	21867787	GGGTGTGGACTTTACGAACCAA	GCACCAAAGGTCATTTGTGGT	172	Same with Ion_AmpliSeq_Fwd_Primer	Same with Ion_AmpliSeq_Rev_Primer
rs2032636	Y	15027529	TGTGACACTGCAATAGTTACTACTTGAG	CAAATCCCATATCCAGCATCCTATCA	168	Same with Ion_AmpliSeq_Fwd_Primer	Same with Ion_AmpliSeq_Rev_Primer
rs2032652	Y	21917313	AGAAGGATGCTGCTCAGCTT	CACTTTGGGTCCAGGATCACC	119	TCCTGGATTCAGCTCTCTTC	AGGATCACCAGCAAAGGTAG
rs2032658	Y	15581983	TCAGAAGAAAGGAAAAATCAGAAGTATCCC	TTCTTTTGCCAATTAGGTCACTTCAAC	157	Same with Ion_AmpliSeq_Fwd_Primer	Same with Ion_AmpliSeq_Rev_Primer
rs2032673	Y	21894058	GAAGGAATCAGCCATTTCACCAAAA	GAAAAATTTATCTCCCCTTAGCTCTCCT	170	Same with Ion_AmpliSeq_Fwd_Primer	Same with Ion_AmpliSeq_Rev_Primer
rs2033003	Y	23550924	GTTGCTCTGTCATCAGGCTGAA	CCTTTCTCTCTAAACTTGAGGCTGAG	141	GCAGGGTGTTGCTCTGTC	TGGCCTCTGGTATCATCT
rs2040411	22	47836412	TGGAATGCCAGTTCTTTTGTGT	AGGGAGATTTTCACCACCAATG	119	GGAATAGCAACATGGGTT	CAACTTGGCTTTAACAGA
rs2046361	4	10969059	CACTACTGGCTCACCTACTATGTATGTA	TCTGCCAAATGTATCCTTACCTTTAAGAC	220	Same with Ion_AmpliSeq_Fwd_Primer	Same with Ion_AmpliSeq_Rev_Primer
rs2056277	8	139399116	GCTTCATTCAATCCGTGAGCAA	CGTTCTGTATAGGCACCATATAGCA	175	Same with Ion_AmpliSeq_Fwd_Primer	Same with Ion_AmpliSeq_Rev_Primer
rs2076848	11	134667546	ATCAGAAACTCCCATGAAACTTTTCAAC	CCAGTGAAATTATTGATAATACACAGGTATCCT	120	CTCACCACCAGAAATCAG	GTTTAACTTGCCTGGAGTA
rs2111980	12	106328254	AGCAAGATCTTTGCCAGTGAGT	GCCATGTTGTAAACATTTTTACGGTCAA	100	TTTACGGTCAAAGCATCT	AACCACTCGTCCAAGAAC
rs214955	6	152697706	ATTCTGGCAGCCCTTGGATG	TCAAACATGATTTTCATCAACTTTATCGCTTT	100	TCTGGCTTGATTAAAGAG	CCAACCACTGCTGGAAAT
rs221956	21	43606997	ATGAATGCTTTCCCTCCAGCTC	GGGTCCATGCTAGAAAAAGCTG	103	CTATAAGCAGTCAGGAGGAA	AGTGGAGGCTGGAAGAGT
rs2269355	12	6945914	CCAGCTCTGCCCCACTTTT	GCTGCTCTTTCCCAGAGAGT	173	Same with Ion_AmpliSeq_Fwd_Primer	Same with Ion_AmpliSeq_Rev_Primer
rs2292972	17	80765788	CCCTGCCGGGTCACGAG	GATGAGGATGTGGAAAAGCAAGGG	75	TCACGAGCAGGCCGGGTTT	AGGATGTGGAAAAGCAAGGG
rs2319818	Y	16354708	TCAATAACAAGTTCTGAAATTAAGGCTGT	GGAATGTTGCCAGCTCCTCTTT	121	ATTCCCACTCCCAAGACT	CTGCTGGATTTGGTTTGC
rs2342747	16	5868700	AAAAGTTGTCAGCATGGGAGGA	TAGCTGCAGGCAGCAGTAAC	115	CCTTCTGAAATCCGTCTC	AGCAGTAACCAGCAACAC
rs251934	5	174778678	AATCCAGACTTAACTACCAGGCA	CAAAGTTATTGCCTAGCCTGAAGTAATTAG	120	TAATTAGAGGGCAGTGAGGC	TCCAGACTTAACTACCAGGC
rs2534636	Y	2657176	CTACAACAGGAAATCCCTTACAAGATGA	GGGATCATTCAGTATCTGGCCTCT	160	Same with Ion_AmpliSeq_Fwd_Primer	Same with Ion_AmpliSeq_Rev_Primer

SNP	Chromosome	Position (GRch37.p10)	Ion_AmpliSeq_Fwd_Primer	Ion_AmpliSeq_Rev_Primer	NGS Library length (bp)	Sanger sequencing_Fwd_Primer	Sanger sequencing_Rev_Primer
rs2830795	21	28608163	GGTAAATAGGAAGGCTGCAGGTT	GCTCTGAATCAGGATGAGCCA	146	TAGATACCCATGAGACTGG	AGTGGTTGGACGACTAAA
rs2831700	21	29679687	GGGACCTATGCTGTGCAGT	TGGCTCCTTCCAACAATTTCCCTA	110	CAGCCTATTATGGAACCT	GAGCAAATGCTATGTGGG
rs321198	7	137029838	CACAGGCTTCAGGTTACCTGTT	CTCCACACTTTATACAGGTGAAATCTGATTAA	99	AGACGTGGCTCTACAAAC	GTAAAGGGACTGGGAAAA
rs338882	5	178690725	GACATCCCAGCCGAAGAATC	GCTTCATTTTTCTCTCCTTCTGTCTC	93	TCTCACCTTCTTTCGTGTGC	AAGAGCTCTGAGAGACATCC
rs35284970	Y	2734854	TGCAGGGCAATAAACCTTGG	GTTTGGTGGCCAGCCTCTTA	93	TCTCCTCTTCTATTGCAGGG	GCAACAGTAAGTCGAATGCC
rs354439	13	106938411	CTGCGTACTGATAAGAGGTGCTT	GGTGTTCTGGTGGCTTCTCTTT	168	Same with Ion_AmpliSeq_Fwd_Primer	Same with Ion_AmpliSeq_Rev_Primer
rs3780962	10	17193346	CTCAAAAACAAAGAAACATGGGATGAACA	TGCGGTAGCGGGCTTTT	96	TTCTGTGACTGGTGGTGT	CTGAAAGTGATGGCAAAT
rs3848982	Y	21717208	CCTCCCACTCCTTTTTGGATCA	GCGGCATACTTGCCTCCA	99	TATAGCGGCATACTTGCCTC	GGTTCTTGATTAGGCTAAGG
rs3900	Y	21730257	CAAGGAATTCGCTGCAGCATA	ATGCATAATGAAGTAAGCGCTACCT	174	Same with Ion_AmpliSeq_Fwd_Primer	Same with Ion_AmpliSeq_Rev_Primer
rs3911	Y	21733454	GTCTTTGTAGATTGGGTGTCTTCAGT	GTCTACATGTTCAGTGCAAATGCAA	175	Same with Ion_AmpliSeq_Fwd_Primer	Same with Ion_AmpliSeq_Rev_Primer
rs4141886	Y	14197867	ATCCCAACTTCGGTTTTTGGTAAAACT	CAAGTAACTATTGTCATCCAAGGAGCA	100	CTTCCACTGGGTGCCTCT	TCTGGTCCCGTCTTTGAT
rs4288409	8	136839229	CCTTGGAGTAAGAAAACCACTGAGA	GCCTGAGGCACACTCAGATG	134	GGCAACAGCCAAAGCATA	TGCCCACATTTCTGACCT
rs430046	16	78017051	AAGGTCATACAATGAATGGTGTGATGT	ACCTATGGGCTCTTCTTATTTCTCCT	100	GGGGAGGGAGTAGAGTGGA	AAGGCAAGAGGAGTCAAGG
rs4364205	3	32417644	CAATACTTGTAACCCAGCAATTCCAG	CCATTTGATAGCCATTTGGGTTGTT	141	AGGTGTTGGCGAGGTTAT	TTTGGTCTGGCTTCTTTT
rs445251	20	15124933	CTCGACCTCCCAAACTGTTAGAATTAA	GCTGCTTGAGTAAACCCATCAC	159	Same with Ion_AmpliSeq_Fwd_Primer	Same with Ion_AmpliSeq_Rev_Primer
rs4530059	14	104769149	ATATGCAGTCGATGATGACTTCCC	CAACGGCTTCCTCAGTAGCAG	100	TGCAGTCGATGATGACTTCC	AGCAACGGCTTCCTCAGTAG
rs4847034	1	105717631	ATTCCTTTGGGTGTAAACCTATGCAG	CTGAGAAGGTAGATTGGTGCAGCTA	118	AGGCAATGTTCTTGGTGT	GAGAAGGTAGATTGGTGC
rs560681	1	160786670	CAACCCTGTCAGCAGGAGTT	AGCCTCTTACTGCACTTCATAAAGAAT	148	TCTGAGAACATCTGTTCAGG	AGGATGCAAACTCTTGGAGG
rs576261	19	39559807	CTCCCCCTCCGTGTACCA	TGTTGATGATAGTGGCAAAGGAGA	75	GTGAACAGCCAGTGGACC	GCCATCCTGGAGCTTCTT
rs6444724	3	193207380	ATTGGAATGGGAGGAAAGGGAAA	TCCTACTCCGTAGTAAATGAGAGC	95	GGATGTTGTTCTGGCTAA	AAGGGAAGGATAATGACT
rs6811238	4	169663615	CCCAGGGAAGGAATAAGTACATTTGAA	CTCAAAGCACCAGGCATTTGAC	162	Same with Ion_AmpliSeq_Fwd_Primer	Same with Ion_AmpliSeq_Rev_Primer
rs6955448	7	4310365	GAAAGAGGAAAGGCATGAATTAAGTTGG	AACAAGACAGGGCAGTTCTTTTCT	135	CCGTGGGCTGTTATCCTG	TCTCCCTTCCGATGTGGC
rs7041158	9	27985938	GCAATGGTGAGAGGTTGATGGT	GAATTTCTGAGAATAACATTGCCTCTCC	170	Same with Ion_AmpliSeq_Fwd_Primer	Same with Ion_AmpliSeq_Rev_Primer
rs717302	5	2879395	ATGGCAAATAAGCTTTAGAAAGGCAT	CACAGAAAGAGGTTCATATGTTGGG	96	TCCCCCAACATATGAACCTC	TCTCCCTCTCTGTTGGTTTGA

SNP	Chromosome	Position (GRch37.p10)	Ion_AmpliSeq_Fwd_Primer	Ion_AmpliSeq_Rev_Primer	NGS Library length (bp)	Sanger sequencing_Fwd_Primer	Sanger sequencing_Rev_Primer
rs719366	19	28463337	AAGGACTTATAGTGAGTAAAGGACAGG	ATCTTTTAACTCTTTTATTATCCTTTCCTGCTTT	96	TCATAAAGGCAAGAACTG	ACTGAGGCAGGGAGATTA
rs722098	21	16685598	CCACCCCTCCCCTATAAACCT	GTTGGGTAAAGAAATATTCAGCACATCC	145	CAACAGCAAGTGCCAGTA	TCCCATTCCCACAGATAG
rs722290	14	53216723	GCACCAAATGACTGTATGAAATTACGTT	AAATGCTGGTCCCAAGGTAGG	173	Same with Ion_AmpliSeq_Fwd_Primer	Same with Ion_AmpliSeq_Rev_Primer
rs727811	6	165045334	ACTTAATATTTCATGCCCACAGAGCA	CTCATGAGCGTTTTCCTCTGTTTTT	165	Same with Ion_AmpliSeq_Fwd_Primer	Same with Ion_AmpliSeq_Rev_Primer
rs729172	16	5606197	TTTATGCCTAAGCCAGCGTGTTA	CTTGGAATTAGCCTTAAAGCAAAGCA	160	Same with Ion_AmpliSeq_Fwd_Primer	Same with Ion_AmpliSeq_Rev_Primer
rs733164	22	27816784	AGCTTTCAGCCCCAGGTC	GCTTGAGTTTTCTTTGGCAAGG	95	CACCAACAGGCCATCCCA	CTACCGAGCCATCCAAGC
rs735155	10	3374178	CTAGTCGGGAGAATGAACGGTCT	GTCGGGAGAGGCCTGCT	100	GCGTAGCTGTAGCTTCCA	CCTCTTGCCTGCTCTTTC
rs737681	7	155990813	GCGGGAGCTCTGCACAA	TGGAAATCCAGAGGCTTCGG	102	AACCACTCTATCTTCATCCCTA	CCTCCCGTTCTGACTTTT
rs740598	10	118506899	CTTTAGAAATGCCTTCTCAGGTAATGGA	CCGGGATGTCCCGTCTTATTAATG	166	Same with Ion_AmpliSeq_Fwd_Primer	Same with Ion_AmpliSeq_Rev_Primer
rs740910	17	5706623	CGCAGCTGTTAAAAAGAATGAGTTAGAT	CCCTTTCTCTGTTCATAGGCAAACA	175	Same with Ion_AmpliSeq_Fwd_Primer	Same with Ion_AmpliSeq_Rev_Primer
rs7520386	1	14155402	GACCAGGACTGTACGTGTGT	TACCCAAGAAGACTCTGTCCCA	100	TGTACGTGTGTCCACCAAGG	TACCCAAGAAGACTCTGTCC
rs7704770	5	159487953	CTGGCTTCAGAGCCCATCTAC	AGGGTTAGTAATCCTGTGGCAAAAT	163	Same with Ion_AmpliSeq_Fwd_Primer	Same with Ion_AmpliSeq_Rev_Primer
rs8179021	Y	15018582	TCTACGGCATAGAAAGTTTGTGCAA	GGCTTTCAGCATAATACCTTACCTAGA	160	Same with Ion_AmpliSeq_Fwd_Primer	Same with Ion_AmpliSeq_Rev_Primer
rs826472	10	2406631	GTATTGTGGAGTTGTTAGGTGTAGCA	CCAGCAAAAACTTCTTTTCTCCAGTA	205	Same with Ion_AmpliSeq_Fwd_Primer	Same with Ion_AmpliSeq_Rev_Primer
rs873196	14	98845531	CTTCAGAATCAGATAGCCCTGCAT	TGGCAGGAGTTGGAGTCAATC	85	TCAAATCCCAAGTGCTGC	TGGAGTCCTGCTCTGTCG
rs876724	2	114974	AATCATCATGCAGGCTCCATTTTT	CTAAATTTGAATATCTATGAGCAGGCAGTT	100	AGCTACCCTGGGAAAGAG	CATTTAAGCAGGTGAAACAT
rs891700	1	239881926	AGAGGTGGTATTTCTAGCTGAGTGT	CTTTCCTTGTGTTTTCTTCAATCACTTCT	164	Same with Ion_AmpliSeq_Fwd_Primer	Same with Ion_AmpliSeq_Rev_Primer
rs901398	11	11096221	AGGACAGCCATTTTGACAACTAC	GGAGCTTAGCTGGGCATCTG	119	GGATGCTGGCTTGTAAGG	CTGCGTGTTCAAATGTTC
rs907100	2	239563579	AGCCCAGGGAGTTCCTGATAA	GTCCTCTTTTGTCTTGGTCTTTGCTA	172	Same with Ion_AmpliSeq_Fwd_Primer	Same with Ion_AmpliSeq_Rev_Primer
rs914165	21	42415929	AATACAAGCAGCAGAGCCTGGA	GGAGTGCAGACCAGTCACC	113	ACGACGAGAATACAAGCA	AGAAGACAGGCAGAATGA
rs917118	7	4457003	CCAACAGAGTCCTCCATGAAGAT	CCAGAGCACAAGTGGTAAGAGAT	84	TCGGTTCCAACAGAGTCCTC	CAGAGCACAAGTGGTAAGAG
rs9341278	Y	15469724	CTGGAAAATGTGGGCTCGTTTT	CAGCAAGTTTATTCGTTGTGTTTGACA	175	Same with Ion_AmpliSeq_Fwd_Primer	Same with Ion_AmpliSeq_Rev_Primer
rs938283	17	77468498	CCCTTCTATTATGGACTGAATCATGCC	CATGTCTGTGTCCTAATCTTCTTCTCT	175	Same with Ion_AmpliSeq_Fwd_Primer	Same with Ion_AmpliSeq_Rev_Primer
rs964681	10	132698419	GCGTCAGGAAGGTACCTGGA	TGGTAAGAACCAGAGGTTGCTG	95	ACCTGGAGGTGATTTCTGTG	AAAGTGCTGGGATTACGG

SNP	Chromosome	Position (GRch37.p10)	Ion_AmpliSeq_Fwd_Primer	Ion_AmpliSeq_Rev_Primer	NGS Library length (bp)	Sanger sequencing_Fwd_Primer	Sanger sequencing_Rev_Primer
rs9786139	Y	6753519	GGAGCCAAAATTGTGCCACTG	TTTGGGTAGATGGCTAGTAATAAGATTGT	120	TGGGCGACAGAGCGAGACT	TGCCACCTTCCAACATCAAGTA
rs9786184	Y	2887824	AGAGAAAACTGGCCACCCTAG	CTTGCTCTTTCCCCAACACG	120	TATGCAAATGCAGAGTGCCC	TCTTGCTCTTTCCCCAACAC
rs987640	22	33559508	CTACTCCACTGTTTGGCATGAACT	GCCCATGCCATCGTCTGTA	114	CACTGTTTGGCATGAACT	ACTTAGCCCTTCCCTGTA
rs9905977	17	2919393	AGGAAAATTCATGAGCTGGTGTC	CTGAGGGACAAAGCTGACAAG	117	TGGTGTCCAAGGAGGGCTG	TGAGGGACAAAGCTGACAAG
rs993934	2	124109213	GAGCAAAGTATTGTGATAACAGTCTCCA	CCAACTAAGTTCATGAATGGGCTAAAA	173	Same with Ion_AmpliSeq_Fwd_Primer	Same with Ion_AmpliSeq_Rev_Primer
rs9951171	18	9749879	TGTTCCTCTGGGATGCAACAT	TTTCTTGTCCCTGCTTTCATGC	100	TTGTTCCTCTGGGATGCAAC	TCTTGTCCCTGCTTTCATGC

Supplementary Table S2-1 Results of control DNA of 9947A by NGS sequencing and Sanger sequencing

SNP No.	HotSpot ID	Chromosome	Position	NGS sequencing							Sanger Sequencing
				Genotype	Coverage	A Reads	C Reads	G Reads	T Reads	F_{MAR} (%)	
1	rs1490413	chr1	4367323	AA	2304	2300	0	4	0	100	A
2	rs7520386	chr1	14155402	GG	681	0	0	681	0	100	G
3	rs4847034	chr1	105717631	AA	1045	1044	0	0	1	100	A
4	rs560681	chr1	160786670	AG	1890	980	0	908	2	52	AG
5	rs10495407	chr1	238439308	AG	1580	843	0	736	1	53	AG
6	rs891700	chr1	239881926	AG	1618	800	1	815	2	50	AG
7	rs1413212	chr1	242806797	CT	1526	0	788	0	738	52	CT
8	rs876724	chr2	114974	CC	883	0	883	0	0	100	C
9	rs1109037	chr2	10085722	AG	1677	873	0	804	0	52	AG
10	rs993934	chr2	124109213	AG	686	319	0	367	0	53	AG
11	rs12997453	chr2	182413259	AG	591	313	0	278	0	53	AG
12	rs907100	chr2	239563579	CC	1135	2	1132	0	1	100	C
13	rs1357617	chr3	961782	TT	1211	1	6	0	1204	99	T
14	rs4364205	chr3	32417644	GT	2445	1	0	1226	1218	50	GT
15	rs1872575	chr3	113804979	AA	2755	2752	0	2	1	100	A
16	rs1355366	chr3	190806108	CC	1434	1	1432	0	1	100	C
17	rs6444724	chr3	193207380	CC	1247	0	1247	0	0	100	C
18	rs2046361	chr4	10969059	AA	1504	1490	0	0	14	99	A
19	rs6811238	chr4	169663615	TT	1903	0	3	0	1900	100	T
20	rs1979255	chr4	190318080	GG	1360	0	1	1359	0	100	G
21	rs717302	chr5	2879395	GG	1777	0	0	1777	0	100	G
22	rs159606	chr5	17374898	AG	1237	655	0	582	0	53	AG

SNP No.	HotSpot ID	Chromosome	Position	NGS sequencing							Sanger Sequencing
				Genotype	Coverage	A Reads	C Reads	G Reads	T Reads	F_{MAR} (%)	
23	rs7704770	chr5	159487953	AA	1318	1317	0	1	0	100	A
24	rs251934	chr5	174778678	AA	1504	1500	0	4	0	100	A
25	rs338882	chr5	178690725	AG	2366	1184	1	1181	0	50	AG
26	rs13218440	chr6	12059954	AG	1762	850	3	909	0	52	AG
27	rs214955	chr6	152697706	TT	617	0	1	0	616	100	T
28	rs727811	chr6	165045334	GG	878	1	0	876	1	100	G
29	rs6955448	chr7	4310365	CT	1453	0	667	1	785	54	CT
30	rs917118	chr7	4457003	CC	1311	0	1311	0	0	100	C
31	rs321198	chr7	137029838	CC	1288	0	1286	0	2	100	C
32	rs737681	chr7	155990813	CC	1320	0	1320	0	0	100	C
33	rs10092491	chr8	28411072	CC	1644	0	1631	0	13	99	C
34	rs4288409	chr8	136839229	AC	854	425	429	0	0	50	AC
35	rs2056277	chr8	139399116	CT	1715	0	881	0	834	51	CT
36	rs1015250	chr9	1823774	GG	780	0	0	780	0	100	G
37	rs7041158	chr9	27985938	CT	1461	0	741	0	720	51	CT
38	rs1463729	chr9	126881448	TT	1684	0	5	1	1678	100	T
39	rs1360288	chr9	128968063	CT	1411	0	780	1	630	55	CT
40	rs10776839	chr9	137417308	GT	1044	0	2	549	493	53	GT
41	rs826472	chr10	2406631	CC	811	0	811	0	0	100	C
42	rs735155	chr10	3374178	CC	1029	0	1028	0	1	100	C
43	rs3780962	chr10	17193346	GG	1352	5	0	1347	0	100	G
44	rs740598	chr10	118506899	AA	1763	1762	0	1	0	100	A
45	rs964681	chr10	132698419	TT	1167	0	0	0	1167	100	T
46	rs1498553	chr11	5709028	TT	2168	0	3	2	2163	100	T

SNP No.	HotSpot ID	Chromosome	Position	NGS sequencing							Sanger Sequencing
				Genotype	Coverage	A Reads	C Reads	G Reads	T Reads	F_{MAR} (%)	
47	rs901398	chr11	11096221	CT	1718	0	908	1	809	53	CT
48	rs10488710	chr11	115207176	CG	1030	0	496	533	1	52	CG
49	rs2076848	chr11	134667546	AT	1486	766	1	2	717	52	AT
50	rs2269355	chr12	6945914	GG	2643	2	1	2640	0	100	G
51	rs2111980	chr12	106328254	CT	989	0	488	1	500	51	CT
52	rs10773760	chr12	130761696	AG	1884	870	0	1014	0	54	AG
53	rs1335873	chr13	20901724	AA	2240	2234	3	2	1	100	A
54	rs1886510	chr13	22374700	AA	1187	1184	2	1	0	100	A
55	rs1058083	chr13	100038233	AG	2368	1288	0	1080	0	54	AG
56	rs354439	chr13	106938411	AT	1444	656	0	0	788	55	AT
57	rs1454361	chr14	25850832	TT	2032	0	2	0	2030	100	T
58	rs722290	chr14	53216723	GG	1245	1	0	1244	0	100	G
59	rs873196	chr14	98845531	TT	1835	0	17	0	1818	99	T
60	rs4530059	chr14	104769149	GG	2036	0	0	2036	0	100	G
61	rs2016276	chr15	24571796	TT	1755	0	0	0	1755	100	T
62	rs1821380	chr15	39313402	CC	1519	0	1518	0	1	100	C
63	rs1528460	chr15	55210705	TT	1378	1	1	0	1376	100	T
64	rs729172	chr16	5606197	GT	1108	0	2	561	545	51	GT
65	rs2342747	chr16	5868700	GG	514	0	0	513	1	100	G
66	rs430046	chr16	78017051	CT	1648	0	864	0	784	52	CT
67	rs1382387	chr16	80106361	AA	1615	1607	0	4	4	100	A
68	rs9905977	chr17	2919393	GG	1562	1	0	1560	1	100	G
69	rs740910	chr17	5706623	GG	1713	2	1	1702	8	99	G
70	rs938283	chr17	77468498	CT	2421	0	1188	0	1233	51	CT

SNP No.	HotSpot ID	Chromosome	Position	NGS sequencing							Sanger Sequencing
				Genotype	Coverage	A Reads	C Reads	G Reads	T Reads	F_{MAR} (%)	
71	rs2292972	chr17	80765788	TT	1700	0	1	0	1699	100	T
72	rs1493232	chr18	1127986	AC	1146	548	598	0	0	52	AC
73	rs9951171	chr18	9749879	AA	954	950	0	3	1	100	A
74	rs1736442	chr18	55225777	CC	1648	0	1647	0	1	100	C
75	rs1024116	chr18	75432386	CT	2503	4	1156	0	1343	54	CT
76	rs719366	chr19	28463337	AG	1238	597	0	641	0	52	AG
**77**	**rs576261**	**chr19**	**39559807**	**AC**	**1615**	**193**	**1422**	**0**	**0**	**88**	**C**
78	rs1031825	chr20	4447483	CC	1797	67	1729	0	1	96	C
79	rs445251	chr20	15124933	CG	1743	1	912	829	1	52	CG
80	rs1005533	chr20	39487110	AG	1479	719	0	760	0	51	AG
81	rs1523537	chr20	51296162	TT	1220	0	3	0	1217	100	T
82	rs722098	chr21	16685598	AA	1741	1739	0	2	0	100	A
83	rs2830795	chr21	28608163	AG	2027	977	1	1049	0	52	AG
84	rs2831700	chr21	29679687	AA	1517	1517	0	0	0	100	A
85	rs914165	chr21	42415929	GG	1252	1	0	1251	0	100	G
86	rs221956	chr21	43606997	CC	2167	0	2162	0	5	100	C
87	rs733164	chr22	27816784	GG	1143	1	0	1142	0	100	G
88	rs987640	chr22	33559508	TT	1614	0	0	0	1614	100	T
89	rs2040411	chr22	47836412	GG	2060	3	0	2057	0	100	G
90	rs1028528	chr22	48362290	GG	1487	0	1	1486	0	100	G
91	rs2534636	chrY	2657176	-	0	0	0	0	0	0	/
92	rs35284970	chrY	2734854	-	0	0	0	0	0	0	/
93	rs9786184	chrY	2887824	-	0	0	0	0	0	0	/
94	rs9786139	chrY	6753519	-	0	0	0	0	0	0	/

SNP No.	HotSpot ID	Chromosome	Position	NGS sequencing							Sanger Sequencing
				Genotype	Coverage	A Reads	C Reads	G Reads	T Reads	F_{MAR} (%)	
95	rs16981290	chrY	7568568	-	0	0	0	0	0	0	/
96	rs17250845	chrY	8418927	-	0	0	0	0	0	0	/
97	L298	chrY	8467290	-	0	0	0	0	0	0	/
98	P256	chrY	8685230	-	0	0	0	0	0	0	/
99	P202	chrY	14001024	-	0	0	0	0	0	0	/
100	rs17306671	chrY	14031334	-	0	0	0	0	0	0	/
101	rs4141886	chrY	14197867	-	0	0	0	0	0	0	/
102	rs2032595	chrY	14813991	-	0	0	0	0	0	0	/
103	rs2032599	chrY	14851554	-	0	0	0	0	0	0	/
104	rs20320	chrY	14898163	-	0	0	0	0	0	0	/
105	rs2032602	chrY	14954280	-	0	0	0	0	0	0	/
106	rs8179021	chrY	15018582	-	0	0	0	0	0	0	/
107	rs2032624	chrY	15026424	-	0	0	0	0	0	0	/
108	rs2032636	chrY	15027529	-	0	0	0	0	0	0	/
109	rs9341278	chrY	15469724	-	0	0	0	0	0	0	/
110	rs2032658	chrY	15581983	-	0	0	0	0	0	0	/
111	rs2319818	chrY	16354708	-	0	0	0	0	0	0	/
112	rs17269816	chrY	17053771	-	0	0	0	0	0	0	/
113	rs17222573	chrY	17891241	-	3	3	0	0	0	0	/
114	M479	chrY	20834667	-	0	0	0	0	0	0	/
115	rs3848982	chrY	21717208	-	0	0	0	0	0	0	/
116	rs3900	chrY	21730257	-	0	0	0	0	0	0	/
117	rs3911	chrY	21733454	-	0	0	0	0	0	0	/
118	rs2032631	chrY	21867787	-	0	0	0	0	0	0	/

SNP No.	HotSpot ID	Chromosome	Position	NGS sequencing							Sanger Sequencing
				Genotype	Coverage	A Reads	C Reads	G Reads	T Reads	F_{MAR} (%)	
119	rs2032673	chrY	21894058	-	0	0	0	0	0	0	/
120	rs2032652	chrY	21917313	-	0	0	0	0	0	0	/
121	rs16980426	chrY	22214221	-	0	0	0	0	0	0	/
122	rs13447443	chrY	22739301	-	1	1	0	0	0	0	/
123	rs17842518	chrY	23443971	-	0	0	0	0	0	0	/
124	rs2033003	chrY	23550924	-	1	0	1	0	0	0	/

Supplementary Table S2-2 Results of control DNA of 9948 by NGS sequencing and Sanger sequencing

SNP No.	HotSpot ID	Chromosome	Position	NGS sequencing							Sanger Sequencing
				Genotype	Coverage	A Reads	C Reads	G Reads	T Reads	F_{MAR} (%)	
1	rs1490413	chr1	4367323	GG	2893	0	0	2893	0	100	G
2	rs7520386	chr1	14155402	AG	1585	1166	0	419	0	74	AG
3	rs4847034	chr1	105717631	GG	1225	7	0	1216	2	99	G
4	rs560681	chr1	160786670	AG	2424	1201	0	1222	1	50	AG
5	rs10495407	chr1	238439308	AG	1964	1000	0	964	0	51	AG
6	rs891700	chr1	239881926	GG	1711	1	0	1709	1	100	G
7	rs1413212	chr1	242806797	CC	1605	0	1604	0	1	100	C
8	rs876724	chr2	114974	CT	865	1	471	0	393	54	CT
9	rs1109037	chr2	10085722	AA	1951	1938	1	12	0	99	A
10	rs993934	chr2	124109213	AA	809	809	0	0	0	100	A
11	rs12997453	chr2	182413259	GG	780	0	0	780	0	100	G
12	rs907100	chr2	239563579	CG	1224	0	606	618	0	50	CG
13	rs1357617	chr3	961782	TT	1398	1	6	0	1391	99	T
14	rs4364205	chr3	32417644	GG	3164	5	0	3153	6	100	G
15	rs1872575	chr3	113804979	AG	3368	1624	1	1743	0	52	AG
16	rs1355366	chr3	190806108	CT	1618	0	832	0	786	51	CT
17	rs6444724	chr3	193207380	CC	1591	2	1586	3	0	100	C
18	rs2046361	chr4	10969059	AA	1754	1745	0	0	9	99	A
19	rs6811238	chr4	169663615	GG	2262	0	0	2262	0	100	G
20	rs1979255	chr4	190318080	GG	1591	0	0	1591	0	100	G
21	rs717302	chr5	2879395	AG	2114	1055	1	1058	0	50	AG
22	rs159606	chr5	17374898	GG	1495	1	0	1494	0	100	G

SNP No.	HotSpot ID	Chromosome	Position	NGS sequencing							Sanger Sequencing
				Genotype	Coverage	A Reads	C Reads	G Reads	T Reads	F_{MAR} (%)	
23	rs7704770	chr5	159487953	GG	1653	0	0	1653	0	100	G
24	rs251934	chr5	174778678	AG	1974	1022	2	948	2	52	AG
25	rs338882	chr5	178690725	AA	2620	2613	2	4	1	100	A
26	rs13218440	chr6	12059954	AG	2313	1140	5	1167	1	50	AG
27	rs214955	chr6	152697706	CT	1062	0	685	0	377	65	CT
28	rs727811	chr6	165045334	TT	963	0	0	0	963	100	T
29	rs6955448	chr7	4310365	CT	1944	0	953	3	988	51	CT
30	rs917118	chr7	4457003	CT	1188	1	660	0	527	56	CT
31	rs321198	chr7	137029838	TT	1253	1	0	0	1252	100	T
32	rs737681	chr7	155990813	CC	1546	0	1542	0	4	100	C
33	rs10092491	chr8	28411072	TT	1983	0	3	0	1980	100	T
34	rs4288409	chr8	136839229	CC	1006	4	1002	0	0	100	C
35	rs2056277	chr8	139399116	TT	1868	0	0	0	1868	100	T
36	rs1015250	chr9	1823774	GG	913	1	0	912	0	100	G
37	rs7041158	chr9	27985938	CT	1759	0	884	0	875	50	CT
38	rs1463729	chr9	126881448	CC	2201	0	2201	0	0	100	C
39	rs1360288	chr9	128968063	CT	1792	0	870	1	921	51	CT
40	rs10776839	chr9	137417308	TT	1166	0	3	14	1149	99	T
41	rs826472	chr10	2406631	CT	862	0	427	0	435	50	CT
42	rs735155	chr10	3374178	CT	1409	0	710	1	698	50	CT
43	rs3780962	chr10	17193346	GG	1539	1	1	1537	0	100	G
44	rs740598	chr10	118506899	AA	1915	1911	0	3	1	100	A
45	rs964681	chr10	132698419	TT	1744	0	1	0	1743	100	T
46	rs1498553	chr11	5709028	CC	2702	0	2701	0	1	100	C

SNP No.	HotSpot ID	Chromosome	Position	NGS sequencing							Sanger Sequencing
				Genotype	Coverage	A Reads	C Reads	G Reads	T Reads	F_{MAR} (%)	
47	rs901398	chr11	11096221	CC	2351	0	2349	1	1	100	C
48	rs10488710	chr11	115207176	CG	1086	0	532	553	1	51	CG
49	rs2076848	chr11	134667546	TT	1654	0	7	1	1646	100	T
50	rs2269355	chr12	6945914	GG	3407	2	0	3405	0	100	G
51	rs2111980	chr12	106328254	TT	1051	0	1	0	1050	100	T
52	rs10773760	chr12	130761696	AG	2055	983	0	1072	0	52	AG
53	rs1335873	chr13	20901724	AA	2628	2623	0	2	3	100	A
54	rs1886510	chr13	22374700	AG	1534	787	0	747	0	51	AG
55	rs1058083	chr13	100038233	GG	2932	3	0	2929	0	100	G
56	rs354439	chr13	106938411	AT	1749	823	0	0	926	53	AT
57	rs1454361	chr14	25850832	AT	2152	1141	1	2	1008	53	AT
58	rs722290	chr14	53216723	GG	1306	0	0	1306	0	100	G
59	rs873196	chr14	98845531	CT	1992	0	1028	0	964	52	CT
60	rs4530059	chr14	104769149	AG	1651	1061	0	590	0	64	AG
61	rs2016276	chr15	24571796	CT	2017	1	991	0	1025	51	CT
62	rs1821380	chr15	39313402	CC	1871	0	1870	0	1	100	C
63	rs1528460	chr15	55210705	TT	1597	1	0	0	1596	100	T
64	rs729172	chr16	5606197	TT	1140	1	2	0	1137	100	T
65	rs2342747	chr16	5868700	AG	548	300	0	248	0	55	AG
66	rs430046	chr16	78017051	TT	1861	0	0	0	1861	100	T
67	rs1382387	chr16	80106361	AC	1833	908	921	2	2	50	AC
68	rs9905977	chr17	2919393	GG	1952	1	0	1949	2	100	G
69	rs740910	chr17	5706623	AG	1885	957	0	921	7	51	AG
70	rs938283	chr17	77468498	CC	2944	0	2940	1	3	100	C

SNP No.	HotSpot ID	Chromosome	Position	NGS sequencing							Sanger Sequencing
				Genotype	Coverage	A Reads	C Reads	G Reads	T Reads	F_{MAR} (%)	
71	rs2292972	chr17	80765788	TT	2257	0	10	0	2247	100	T
72	rs1493232	chr18	1127986	AA	1378	1301	73	4	0	94	A
73	rs9951171	chr18	9749879	AG	1213	557	0	656	0	54	AG
74	rs1736442	chr18	55225777	CC	1764	0	1764	0	0	100	C
75	rs1024116	chr18	75432386	CT	2519	2	1070	1	1446	57	CT
76	rs719366	chr19	28463337	AG	1318	629	0	688	1	52	AG
77	rs576261	chr19	39559807	AA	1957	1957	0	0	0	100	A
78	rs1031825	chr20	4447483	AC	2521	1326	1190	5	0	53	AC
79	rs445251	chr20	15124933	CG	2068	1	1008	1059	0	51	CG
80	rs1005533	chr20	39487110	AA	1584	1583	0	0	1	100	A
81	rs1523537	chr20	51296162	TT	1339	4	2	0	1333	100	T
82	rs722098	chr21	16685598	AA	2166	2163	0	3	0	100	A
83	rs2830795	chr21	28608163	AA	2303	2296	0	7	0	100	A
84	rs2831700	chr21	29679687	AA	1612	1612	0	0	0	100	A
85	rs914165	chr21	42415929	GG	1539	0	1	1538	0	100	G
86	rs221956	chr21	43606997	CC	2630	3	2625	0	2	100	C
87	rs733164	chr22	27816784	GG	1618	0	1	1617	0	100	G
88	rs987640	chr22	33559508	AT	2148	1055	2	1	1090	51	AT
89	rs2040411	chr22	47836412	AA	2429	2426	0	2	1	100	A
90	rs1028528	chr22	48362290	AG	1835	941	0	894	0	51	AG
91	rs2534636	chrY	2657176	C	768	0	768	0	0	100	C
92	rs35284970	chrY	2734854	C	907	0	907	0	0	100	C
93	rs9786184	chrY	2887824	A	1370	1370	0	0	0	100	A
94	rs9786139	chrY	6753519	G	694	0	0	694	0	100	G

SNP No.	HotSpot ID	Chromosome	Position	NGS sequencing							Sanger Sequencing
				Genotype	Coverage	A Reads	C Reads	G Reads	T Reads	F_{MAR} (%)	
95	rs16981290	chrY	7568568	C	941	0	941	0	0	100	C
96	rs17250845	chrY	8418927	G	1049	0	0	1049	0	100	G
97	L298	chrY	8467290	T	952	0	0	0	952	100	T
98	P256	chrY	8685230	G	836	0	0	836	0	100	G
99	P202	chrY	14001024	T	785	0	0	0	785	100	T
100	rs17306671	chrY	14031334	T	1053	0	2	0	1051	100	T
101	rs4141886	chrY	14197867	A	720	720	0	0	0	100	A
102	rs2032595	chrY	14813991	T	579	0	0	0	579	100	T
103	rs2032599	chrY	14851554	T	721	0	0	0	721	100	T
104	rs20320	chrY	14898163	G	794	0	0	794	0	100	G
105	rs2032602	chrY	14954280	T	1061	0	0	0	1061	100	T
106	rs8179021	chrY	15018582	C	776	0	776	0	0	100	C
107	rs2032624	chrY	15026424	C	722	0	722	0	0	100	C
108	rs2032636	chrY	15027529	G	1046	0	0	1040	6	99	G
109	rs9341278	chrY	15469724	G	581	0	0	581	0	100	G
110	rs2032658	chrY	15581983	G	938	0	0	938	0	100	G
111	rs2319818	chrY	16354708	G	1387	0	0	1387	0	100	G
112	rs17269816	chrY	17053771	C	951	1	949	0	1	100	C
113	rs17222573	chrY	17891241	A	1328	1323	0	4	1	100	A
114	M479	chrY	20834667	C	425	1	424	0	0	100	C
115	rs3848982	chrY	21717208	C	952	1	951	0	0	100	C
116	rs3900	chrY	21730257	G	1508	0	0	1508	0	100	G
117	rs3911	chrY	21733454	A	754	753	0	1	0	100	A
118	rs2032631	chrY	21867787	A	604	603	0	1	0	100	A

SNP No.	HotSpot ID	Chromosome	Position	NGS sequencing							Sanger Sequencing
				Genotype	Coverage	A Reads	C Reads	G Reads	T Reads	F_{MAR} (%)	
119	rs2032673	chrY	21894058	T	972	2	0	0	970	100	T
120	rs2032652	chrY	21917313	T	1006	0	0	0	1006	100	T
121	rs16980426	chrY	22214221	T	1039	0	0	0	1039	100	T
122	rs13447443	chrY	22739301	A	986	985	0	0	1	100	A
123	rs17842518	chrY	23443971	G	1221	0	0	1221	0	100	G
124	rs2033003	chrY	23550924	C	931	0	931	0	0	100	C

Supplementary Table S3 Inconsistent results between the first and second time of NGS sequencing among 45 individuals

Sample name	Chromosome	Position	HotSpot ID	Experiment	Genotype	Coverage	A Reads	C Reads	G Reads	T Reads	F_{MAR} (%)
78#	chr1	14155402	rs7520386	NGS-1	AG	821	649	0	172	0	79.05
				NGS-2	AA	967	893	0	73	1	92.35
				Sanger	A						
A12_045	chr6	152697706	rs214955	NGS-1	CC	70	0	64	0	6	91.43
				NGS-2	CT	120	0	87	0	33	72.50
				Sanger	CT						

Supplementary Table S4 SNPs observed with imbalance of heterozygotes sequenced on PGM platform among 45 individuals

SNP	Minimum F_{MAR} (%)	Maximum F_{MAR} (%)	Mean F_{MAR} (%)	Std. Deviation
rs7520386	64	77	71.21	3.78
rs4530059	51	74	61.44	7.82
rs214955	51	70	58.45	4.44
rs1523537	52	66	56.94	3.47
rs2342747	50	65	54.30	3.94
rs576261	50	62	52.98	2.79

Supplementary Table S5 Genotyping results of 34 Y-SNPs of different ratios of mixtures (9947A:9948)

SNP	9947A	9948	A(100:1)	B(10:1)	C(5:1)	D(1:1)	E(1:5)	F(1:10)	G(1:100)
rs2534636	-	C	-	C	C	C	C	C	C
rs35284970	-	C	-	C	C	C	C	C	C
rs9786184	-	A	-	A	A	A	A	A	A
rs9786139	-	G	-	G	G	G	G	G	G
rs16981290	-	C	C	C	C	C	C	C	C
rs17250845	-	G	-	G	G	G	G	G	G
L298	-	T	-	T	T	T	T	T	T
P256	-	G	-	G	G	G	G	G	G
P202	-	T	-	T	T	T	T	T	T
rs17306671	-	T	-	T	T	T	T	T	T
rs4141886	-	A	-	A	A	A	A	A	A
rs2032595	-	T	-	T	T	T	T	T	T
rs2032599	-	T	-	T	T	T	T	T	T
rs20320	-	G	-	G	G	G	G	G	G
rs2032602	-	T	T	-	T	T	T	T	T
rs8179021	-	C	-	C	C	C	C	C	C
rs2032624	-	C	C	C	C	C	C	C	C
rs2032636	-	G	-	G	G	G	G	G	G
rs9341278	-	G	-	G	G	G	G	G	G
rs2032658	-	G	-	G	G	G	G	G	G
rs2319818	-	G	-	G	G	G	G	G	G
rs17269816	-	C	-	C	C	C	C	C	C
rs17222573	-	A	A	A	A	A	A	A	A

SNP	9947A	9948	A(100:1)	B(10:1)	C(5:1)	D(1:1)	E(1:5)	F(1:10)	G(1:100)
M479	-	C	-	C	C	C	C	C	C
rs3848982	-	C	-	C	C	C	C	C	C
rs3900	-	G	G	G	G	G	G	G	G
rs3911	-	A	-	A	A	A	A	A	A
rs2032631	-	A	-	A	A	A	A	A	A
rs2032673	-	T	-	T	T	T	T	T	T
rs2032652	-	T	-	T	T	T	T	T	T
rs16980426	-	T	-	T	T	T	T	T	T
rs13447443	-	A	-	A	A	A	A	A	A
rs17842518	-	G	-	G	G	G	G	G	G
rs2033003	-	C	-	C	C	C	C	C	C

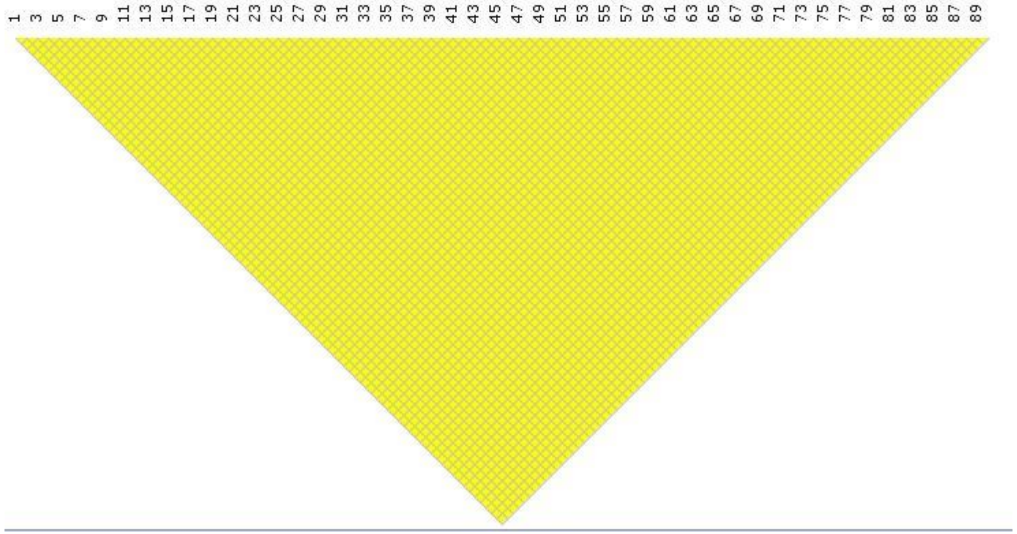

Supplementary Fig S1 LD analysis of 90 auto-SNPs among Chinese HAN population (N=45) by SNP Analyzer 2.0 software. Color of yellow means no LD while red indicated LD existed among the neighbor SNPs.
